# Dietary Supplementation With Creatine Pyruvate Alters Rumen Microbiota Protein Function in Heat-Stressed Beef Cattle

**DOI:** 10.3389/fmicb.2021.715088

**Published:** 2021-08-27

**Authors:** Yanjiao Li, Yitian Zang, Xianghui Zhao, Lin Liu, Qinghua Qiu, Kehui Ouyang, Mingren Qu

**Affiliations:** Jiangxi Province Key Laboratory of Animal Nutrition/Animal Nutrition and Feed Safety Innovation Team, College of Animal Science and Technology, Jiangxi Agricultural University, Nanchang, China

**Keywords:** beef cattle, creatine pyruvate, heat stress, 16S rDNA sequencing, metaproteomics, rumen microbiota protein

## Abstract

Creatine pyruvate (CrPyr) is a new multifunctional nutrient that can provide both pyruvate and creatine. It has been shown to relieve the heat stress of beef cattle by improving antioxidant activity and rumen microbial protein synthesis, but the mechanism of CrPyr influencing rumen fermentation remains unclear. This study aimed to combine 16S rDNA sequencing and metaproteomics technologies to investigate the microbial composition and function in rumen fluid samples taken from heat-stressed beef cattle treated with or without 60 g/day CrPyr. 16S rDNA sequencing revealed that there were no significant differences in the α-diversity indices between the two groups. By analyzing the level profiles of 700 distinct proteins, we found that the CrPyr administration increased the expression of enzymes involved in specific metabolic pathways including (i) fatty acid β-oxidation; (ii) interconversion from pyruvate to phosphoenolpyruvate, oxaloacetate, acetyl-CoA, and malate; (iii) glycolysis/gluconeogenesis and citrate cycle metabolism; and (iv) biosynthesis of amino acids. These results indicated that the increased generation of adenosine triphosphate during fatty acid β-oxidation or citrate cycle and the up-regulation synthesis of microbial protein in rumen of beef cattle treated with CrPyr may help decrease oxidative stress, regulate energy metabolism, and further improve the rumen fermentation characteristic under heat stress.

## Introduction

Ruminal microbiota is a large systematic microbial ecosystem that is composed of bacteria, archaea, protozoa, and fungi (Hobson and Stewart, 1997). Microbial fermentation of feedstuff in the rumen produces volatile fatty acids (VFA) and microbial protein to provide the bulk of the energy and protein required by the host animal (Thomas et al., 2017). Hence, rumen microbes are essential for ruminant growth and health. However, many studies show that heat stress leads to severe defects in rumen function and variation in rumen microbial abundance and diversity (Tajima et al., 2007; Salles et al., 2010; Uyeno et al., 2010). Uyeno et al. (2010) reported that high temperature and humidity increased the abundance of *Streptococcus* and decreased the abundance of *Fibrobacter* and *Oscillospira.* Sales et al. (2021) showed that beef cattle under heat stress presented lower prokaryote richness, genera *Flavonifractor*, *Treponema*, and *Ruminococcus*, higher genera *Carnobacterium*. Moreover, the ruminal pH usually decreases under heat stress conditions (Tajima et al., 2007). A drop in ruminal pH often depressed cellulolytic ruminal bacterial growth and fiber digestion, promoted the cytolysis of gram-negative bacteria, and released lipopolysaccharides, further leading to inflammatory response on the gastrointestinal tract (Dai et al., 2017). Especially in southern China, due to the hot weather and high humidity, feedlot beef cattle finished in the summer months often suffer from heat stress. Therefore, the possibility of controlling the rumen microbial metabolism to achieve more efficient nutrient utilization under heat stress conditions has become an appealing concept for nutritionists.

Manipulation of feed additives such as antibiotics and vitamins is an efficient tool for relieving the negative impact of heat stress. However, a possible linkage between antibiotics in animal feed and the transmission of antimicrobial-resistant bacteria to humans has prompted researchers to explore other alternative approaches. As a new multifunctional nutrient, creatine pyruvate (CrPyr, C_7_H_13_N_3_O_5_, 219.20) contains pyruvic acid and creatine at a ratio of 40:60 (Chen et al., 2012), which both are natural body intermediate metabolites. This means that CrPyr can be used safely in the feed. In heat-stressed ruminants, the reduction of energy supply for microbial growth results in the decreased generation of microbial crude protein (MCP; Hyder et al., 2017). Pyruvate is the intermediate product of carbohydrate fermentation by rumen microorganisms (Nagaraja, 2016). The adenosine triphosphate (ATP) yield in the net conversion of pyruvate to VFA can promote microbial growth. Creatine represents one of the most important nitrogen-containing compounds in protein and energy metabolism and can provide a source of nitrogen for microbial protein synthesis (Navrátil et al., 2009). Thus, we hypothesized that CrPyr may enhance the synchronization of energy and nitrogen supply and be beneficial for ruminal characteristics and microbial growth, and our previous study has shown that dietary supplementation with CrPyr improved rumen microbial protein synthesis. Creatine and pyruvate are not only essential substrates for energetics but also critical antioxidants (Navrátil et al., 2009; Nagaraja, 2016; Rieger et al., 2017). The results in our previous study showed that dietary supplementation with CrPyr improved stress resistance and increased the antioxidant status and rumen fermentation of heat-stressed beef cattle (Liu et al., 2020). It is implied that supplementing with CrPyr could be an effective option to mitigate heat stress suffered by beef cattle. However, to date, detailed information about the changes in rumen microbial communities and related metabolic enzymes associated with the effects of CrPyr on rumen fermentation has not yet been published. Herein, an intensive study of rumen microbial diversity and microbial protein synthesis helps clarify the regulatory mechanism of CrPyr on rumen fermentation.

With the rapid development of bioinformatics, more researchers are starting to use meta-omics technology to define the rumen ecosystem’s ecology and its responses to changes in diet and rearing conditions (Gruninger et al., 2019). Previous studies have shown that 16S rDNA sequencing was employed to characterize the composition and diversity of rumen microbes in Holstein heifers at elevated environmental temperatures and humidity, but with little information about their function (Uyeno et al., 2010; Sales et al., 2021), while metaproteomics can provide the means to study the role of key enzymes involved in ruminant feed utilization efficiency under a particular set of conditions that dictate function. Analysis of the metaproteomes of the rumen microbial community would reveal details about microbial community activity, structure, function, and metabolic pathway transformations that are presently lacking (Hart et al., 2018). Therefore, in this study, we combined 16S rDNA sequencing and metaproteomics technologies to understand the regulatory mechanism of CrPyr on rumen fermentation deeply.

## Materials and Methods

This experiment was approved by the Committee for the Care and Use of Experimental Animals at Jiangxi Agricultural University (JXAULL-20190017).

### Animal Treatments and Experimental Diets

Six ruminally cannulated Jinjiang steers (initial body weight = 405 ± 21 kg) were randomly allocated into two treatments. Jinjiang cattle are a breed of Chinese indigenous beef cattle bred in the northwest of the Jiangxi Province. The Jinjiang cattle received a CrPyr free basal diet (control group, CG) or a 60 g/day CrPyr supplemental basal diet (experiment group, EG) for 22 days (2019/08/02–2019/08/23). The purity of CrPyr is 99.9% (Hubei Ju Sheng Technology Co., Ltd., Wuhan, China). Our previous study results indicated that the Jinjiang cattle fed with 60 g/day CrPyr had a better resistance to heat stress (Liu et al., 2020). Therefore, the present experiment selected a 60-g/day dose to explore the regulatory mechanism of CrPyr on rumen fermentation. CrPyr was added to the concentrates, divided into two daily feeds (07:00 and 16:00). After the concentrate was fed, rice straw was given. All steers were housed in individual solid concrete floor pens in a closed cowshed, while clean, fresh water was available at all times. The composition and nutrient levels of the experimental diet were shown in Supplementary Table 1.

### Measurement of Temperature and Humidity Index, Body Temperature, and Respiratory Rate

The temperature and humidity index (THI) was measured according to Song et al. (2014). The thermohygrometer was suspended on the wall around the cattle’s bodies on both sides of the experimental herd (approximately 1.5 m above the ground). The temperature and relative humidity at 08:00, 14:00, and 20:00 were recorded each day. Calculation of the average daily THI and the Livestock Weather Safety Index (LWSI) classifications for heat stress used the method of LCI (LCI, 1970). The feed volume and 2-h postprandial residual feed volume of each cattle per day were recorded accurately to calculate the average dry matter intake (ADMI = total dry matter intake per cattle/22 days). Body temperature was measured *via* the rectum using a veterinary thermometer at 08:00, and their respiratory rate was measured *via* manual count at 08:30 and 14:00 until day 21 during the experimental period.

### Sample Collection

Samples of feed, feces, and rumen fluid were collected. All feces samples were collected from each steer on days 19 to 21. The feces were collected using a plastic bucket placed behind the cattle. The feces were thoroughly mixed, and 5% of the total feces were sampled once daily. After collection, the 3 days of fecal samples were mixed, and a subsample (300 *g*) was frozen at –20°C and used later to measure dry matter, ash, crude fat, neutral detergent fiber, and acid detergent fiber contents. The other subsample (100 *g*) was thoroughly mixed with 20 ml of 10% concentrated sulfuric acid and stored at –20°C until it could be analyzed for crude protein.

On day 21, rumen fluid samples were collected from the upper, middle, and lower sites in the rumen immediately post-feeding in the morning. Approximately 100 ml of rumen fluid was collected from each collection site. The rumen fluid was immediately measured for pH using a portable pH meter (HANNA Instruments, Cluj-Napoca, Romania). The rumen fluid samples were then filtered through four cheesecloth layers, and the samples were divided into three portions. The first 8 ml of rumen fluid was mixed with 2 ml of 25% (wt/vol) metaphosphoric acid and used for VFA analysis. One subsample (10 ml) of rumen fluid was mixed with 2 ml of H_2_SO_4_ (1% vol/vol) for determination of NH_3_-N, and another subsample (10 ml) of rumen fluid was used for MCP and 16S rDNA sequencing and meta-proteomics analysis. These samples were frozen at –20°C until analysis.

### Chemical Analyses

The VFA concentrations in the rumen fluid samples were determined using gas chromatography (Shimadzu GC-2014, Japan) equipped with a capillary column (Stabilwax, Restek, Bellefonte, PA, United States). The NH_3_-N concentration was measured using a TU-1901 spectrophotometer (Beijing Purkinje General Instrument Co. Ltd., China) according to a method described by Broderick and Kang (Broderick and Kang, 1980). The MCP production was determined according to the method of Makkar and Becker (1999).

The feed and feces samples were dried at 60°C and ground through a 1-mm stainless steel screen. The dry matter, ash, and crude fat of the feed and fecal samples were determined according to methods 967.03, 924.05, and 920.39 of the AOAC (Cunniff, 1995), respectively. The neutral detergent fiber and acid detergent fiber were analyzed using an Ankom A200i Fiber Analyzer (ANKOM Technology Co., New York, NY, United States) according to the methods of Van Soest et al. (1991). The total nitrogen contents of the feed and feces samples were determined according to procedure 984.13 of the AOAC (Cunniff, 1995).

### 16S rDNA Sequencing Analysis

#### DNA Extraction and PCR Amplification

According to the manufacturer, microbial community genomic DNA was extracted from ruminal fluid samples using the E.Z.N.A.^®^ soil DNA Kit (Omega Bio-Tek, Norcross, GA, United States). The DNA extract was checked on 1% agarose gel, and DNA concentration and purity were determined with NanoDrop 2000 UV-vis spectrophotometer (Thermo Scientific, Wilmington, United States). The hypervariable region V3–V4 of the bacterial 16S rRNA gene was amplified with primer pairs 338F (5′-ACTCCTACGGGAGGCAGCAG-3′) and 806R (5′-GGACTACHVGGGTWTCTAAT-3′) by an ABI GeneAmp^®^ 9700 PCR thermocycler (ABI, CA, United States). The PCR amplification of the 16S rRNA gene was performed as follows: initial denaturation at 95°C for 3 min, followed by 27 cycles of denaturing at 95°C for 30 s, annealing at 55°C for 30 s, and extension at 72°C for 45 s, and single extension at 72°C for 10 min, and end at 4°C. The PCR mixtures contain 5 × *TransStart* FastPfu buffer 4 μl, 2.5 mM dNTPs 2 μl, forward primer (5 μM) 0.8 μl, reverse primer (5 μM) 0.8 μl, *TransStart* FastPfu DNA Polymerase 0.4 μl, template DNA 10 ng, and finally ddH_2_O up to 20 μl. PCR reactions were performed in triplicate. The PCR product was extracted from 2% agarose gel and purified using the AxyPrep DNA Gel Extraction Kit (Axygen Biosciences, Union City, CA, United States) according to the manufacturer’s instructions and quantified using Quantus^TM^ Fluorometer (Promega, United States).

#### Illumina MiSeq Sequencing

Purified amplicons were pooled in equimolar and paired-end sequenced (2 × 300) on an Illumina MiSeq platform (Illumina, San Diego, United States) according to the standard protocols by Majorbio Bio-Pharm Technology Co. Ltd. (Shanghai, China). The raw reads were deposited into the NCBI BioProject database (Accession Number: PRJNA717402).

#### Processing of Sequencing Data

The raw 16S rDNA gene sequencing reads were demultiplexed, quality-filtered by Trimmomatic, and merged by FLASH with the following criteria. (i) The 300-bp reads were truncated at any site receiving an average quality score of <20 over a 50-bp sliding window, and the truncated reads shorter than 50 bp were discarded; reads containing ambiguous characters were also discarded. (ii) Only overlapping sequences longer than 10 bp were assembled according to their overlapped sequence. The maximum mismatch ratio of overlap region is 0.2. Reads that could not be assembled were discarded. (iii) Samples were distinguished according to the barcode and primers, and the sequence direction was adjusted, exact barcode matching, two-nucleotide mismatch in primer matching.

Operational taxonomic units (OTUs) with 97% similarity cutoff were clustered using UPARSE (version 7.1, http://drive5.com/uparse/), and chimeric sequences were identified and removed. The taxonomy of each OTU representative sequence was analyzed by RDP Classifier^1^ against the 16S rRNA database (e.g., Silva 132/16s_bacteria) using a confidence threshold of 0.7.

### Meta-Proteomics Analysis

#### Protein Extraction

The processed rumen fluid sample was centrifuged at 4°C for 5 min at 800 *g* to collect the supernatant. The supernatant was centrifuged at 7,000 *g* for 20 min at 4°C, then borax/PVPP/phenol (BPP: 100 mM EDTA, 50 mM borax, 50 mM Vitamin C, 30% sucrose w/v, 100 mM Tris Base, 1% Triton X-100 v/v, and 5 mM DTT, pH 8.0) was added in the ratio of 1:10 to the resultant precipitate and ground with liquid nitrogen three times, and each grind lasted for 120 s. The solution was centrifuged at 12,000 *g* for 20 min at 4°C, and the supernatant was collected. The equal volume of Tris-saturated phenol was added and vortexed for 10 min at 4°C. The solution was centrifuged at 12,000 *g* for 20 min at 4°C, and the phenol phase was collected. The equal volume of BPP was added and vortexed for 10 min at 4°C. The solution was centrifuged at 12,000 *g* for 20 min at 4°C, and the phenol phase was collected. Five volumes of precooled 0.1 M ammonium acetate in methanol were added, and the protein was precipitated at –20°C overnight. The supernatant was discarded by centrifugation, and the precipitate was washed twice with 90% acetone. The supernatant by was discarded by centrifugation, and the precipitate was air-dried. The precipitate was resuspended with lysis buffer (1% SDS, 8 M urea, protease inhibitor cocktail), then sonicated for 2 min on ice. The lysates were centrifuged at 12,000 *g* for 20 min at 4°C, and supernatants were collected to test the protein concentration in all samples. Protein concentrations were determined by the bicinchoninic acid method.

#### Protein Digestion

Protein digestion was performed according to the standard procedure. Briefly, for each sample tube containing 100 μg protein, appropriate triethylammonium bicarbonate (TEAB) buffer was added to the final concentration of 100 mM. Then, Bond-Breaker^TM^ TCEP solution (TCEP) was added to the final concentration of 10 mM, and the tubes were incubated at 37°C for 60 min. Appropriate iodoacetamide was added to the final concentration of 40 mM and reaction for 40 min in the dark. Six volumes of cold acetone were added to the sample tube, and the tube was incubated at –20°C for 4 h. The acetone was removed by centrifugation at 10,000 *g* for 20 min, and precipitated protein was resuspended with 100 μl 100 mM TEAB buffer. To each sample tube, according to the 1:50 proportion, the trypsin solution was added and the tubes were incubated at 37°C overnight.

#### Peptide Desalination and Quantification

The peptides were vacuum dried, then resuspended with 0.1% trifluoroacetic acid. Samples were desalted with an Oasis^®^ HLB 96-well 300-μm plate and vacuum dried. Peptide concentrations were determined by the Thermo Fisher Scientific peptide quantification kit (Thermo, Cat. 23275). The loading buffer was added to each tube to prepare samples for mass spectrometry analysis, and the concentration of each sample was 0.25 μg/μl.

### Mass Spectrometry Analysis

Experiments were performed on a Q Exactive HF-X mass spectrometer that was coupled with Easy-nLC 1200. Each peptide sample was injected for nanoLC-MS/MS analysis. The sample was loaded onto a C18 reversed-phase column (75 μm × 25 cm, Thermo, United States) in buffer A (2% acetonitrile and 0.1% formic acid) and separated with a linear gradient of buffer B (80% acetonitrile and 0.1% formic acid) at a flow rate of 300 nl/min. The electrospray voltage of 1.8 kV vs. the inlet of the mass spectrometer was used. A Q Exactive HF-X mass spectrometer was operated in data-dependent mode to switch automatically between MS and MS/MS acquisition. Survey full-scan MS spectra (m/z 350–1,300) were acquired with a mass resolution of 70 K, followed by 20 sequential high-energy collisional dissociation MS/MS scans with a resolution of 17.5 K. In all cases, one microscan was recorded using the dynamic exclusion of 30 s. The mass spectrometry proteomics data have been deposited to the ProteomeXchange Consortium^2^ *via* the iProX partner repository (Ma et al., 2019) with the dataset identifier PXD025118.

### Taxonomic Analysis of Peptides and Identification of Proteins

MS/MS spectra were searched using Proteome Discoverer^TM^ Software 2.2 software against the UniProt database (uniprot-taxonomy_3A171549_3A186802_3A191303_.fasta/LL_PEAKS_ exported_proteins.fasta) as the following parameters. The highest score for a given peptide mass (best match to that predicted in the database) was used to identify parent proteins. The parameters for protein searching were set as follows: tryptic digestion with up to two missed cleavages, carbamidomethylation of cysteines as fixed modification, and oxidation of methionines and protein N-terminal acetylation as variable modifications. Peptide spectral matches were validated based on *q*-values at a 1% false discovery rate.

### Informatics Protocol

The protein quantification and calculation of statistical significance were carried out using the *t*-test in the *R* language. To perform functional analysis, the identified proteins were carried on the follow-up biological information function analysis using GO (Gene Ontology, http://www.geneontology.org/). Pathway mapping of identified proteins was performed using the KEGG database (Kyoto Encyclopedia of Genes and Genomes, http://www.genome.jp/kegg/).

### Statistical Analysis

Statistical analysis of body temperature, respiratory rate, rumen fermentation, nutrient digestibility, and taxonomy was first tested for Gaussian distribution using the Shapiro–Wilk test and performed by independent sample *T*-test with SPSS (SPSS 2008) software. Mean values and standard error (SE) were reported, and differences were considered to be significant at *P* < 0.05.

## Results

### Temperature and Humidity Index

**FIGURE 1:**
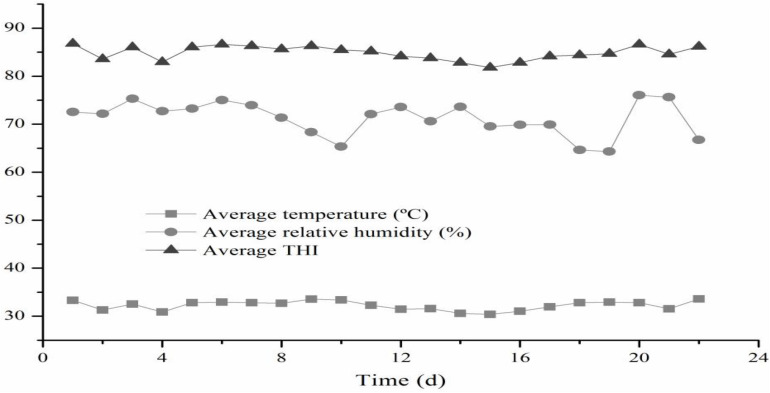
Temperature-humidity index in the house of the beef cattle. THI, temperature and humidity index.

As shown in Figure 1, there were three times daily changes in THI values in the morning (08:00), noon (14:00), and evening (20:00) during the experimental period. The average daily THI values during the experimental period were higher than 79 for all 22 experimental days.

### Average Dry Matter Intake, Body Temperature, Respiratory Rate, Rumen Fermentation, and Nutrient Digestibility

**FIGURE 2:**
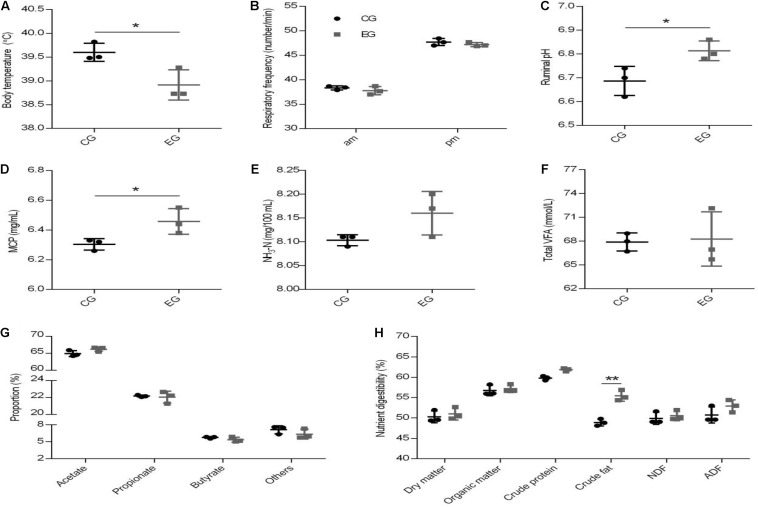
Effect of creatine pyruvate on body temperature **(A)**, respiratory frequency **(B)**, rumen fermentation parameter **(C–G)**, and nutrient digestibility **(H)** of beef cattle. The rumen fermentation parameter including ruminal pH **(C)**, the concentration of microbial crude protein [MCP; **(D)**, NH_3_-N **(E)**, and total VFA **(F)**, the proportion of individual VFA **(G)**. CG, control group; EG, experimental group, diet supplemented with 60 g/day CrPyr. *n* = 3 per group. *P < 0.05, **P < 0.01.

There was no difference in the ADMI between the EG and CG groups (4.89 kg vs. 4.89 kg, *P* = 0.081). Dietary supplementation with CrPyr decreased the body temperature of beef cattle (Figure 2A, *P* < 0.05), but did not affect the respiratory rate of beef cattle (Figure 2B). Diet supplemented with CrPyr increased the ruminal pH value (Figure 2C, *P* < 0.05) and MCP concentration (Figure 2D, *P* < 0.05). No effect was observed on the contents of NH_3_-N (Figure 2E), total VFA (Figure 2F), or individual VFA (Figure 2G). As shown in Figure 2H, CrPyr supplementation increased the crude fat digestibility (*P* < 0.05). No difference was found between groups for the digestibility of the dry matter, organic matter, crude protein, neutral detergent fiber, and acid detergent fiber.

### Diversity of Rumen Fluid Microbiota of Beef Cattle as Revealed by 16S rDNA High-Throughput Sequencing

The high-throughput sequencing technology was used to investigate the effects of CrPyr on microbial communities in the rumen of beef cattle. The sequences were clustered into 1,306 OTUs with a similarity of 97%. Among these, a total of 1,098 OTUs were common, 109 OTUs were specific for the EG, and the CG had 99 unique OTUs (Supplementary Figure 1). The rumen microbiota α-diversity of the two groups was evaluated by the Ace index, Chao1 index, Shannon index, and Simpson index (Supplementary Table 2). According to the result, there were no significant differences in the α-diversity index between the two groups.

### Metaproteomics Analyses of Proteins in the Rumen Fluid Microbiota of Beef Cattle

**FIGURE 3:**
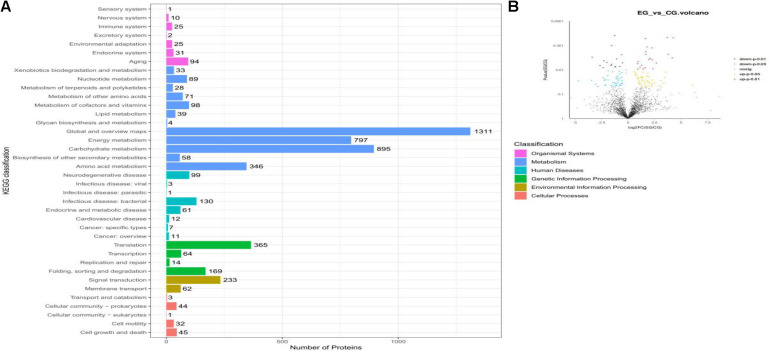
Metaproteomics analyses of proteins in the rumen fluid microbiota of beef cattle fed a CrPyr supplementation diet. **(A)** KEGG classification. **(B)** Volcano plots of differentially expressed proteins between EG and CG. EG, experimental group, diet supplemented with 60 g/day CrPyr; CG, control group. Each dot represents the mean expression level (*n* = 3) of a single protein. The yellow dots indicate the significantly up-regulated proteins at the *P* < 0.05 level, the red dots indicate the significantly up-regulated proteins at the *P* < 0.01 level, the light blue dot indicates the significantly down-regulated proteins at the *P* < 0.05 level, and the blue dot indicates the significantly down-regulated proteins at the *P* < 0.01 level.

Label-free quantification proteomics was applied to investigate the rumen fluid microbiota proteins of beef cattle. A total of 716,150 MS/MS spectra were generated from the six rumen fluid samples. Of these, 11,361 peptides (16.2%) could be identified and assigned to 3,579 proteins (Supplementary Table 3). Of the proteins identified, most were related to cell metabolism (3,407). Others were related to organismal systems (188), human diseases (324), genetic information processing (612), environmental information processing (295), and cellular processes (125; Figure 3A). They included 2,997 proteins that were quantified in the rumen fluid microbiota of EG and CG. The number of overlapping proteins between the two groups was 2,153 (71.8%), while 373 and 206 community-specific proteins were unique to the rumen fluid microbiota in EG and CG, respectively, the number of quantified proteins without quantitative value (refers to a protein expressed in samples with less than two-thirds in both groups) was 265. Furthermore, in the 2,153 overlapping proteins, 121 differentially expressed proteins (DEPs) were identified in the EG compared with the CG, of which 67 proteins were up-regulated and 54 proteins were down-regulated (Figure 3B, *P* < 0.05).

### Composition of Rumen Fluid Microbiota of Beef Cattle as Revealed by 16S rDNA Sequencing and Metaproteomics

**FIGURE 4:**
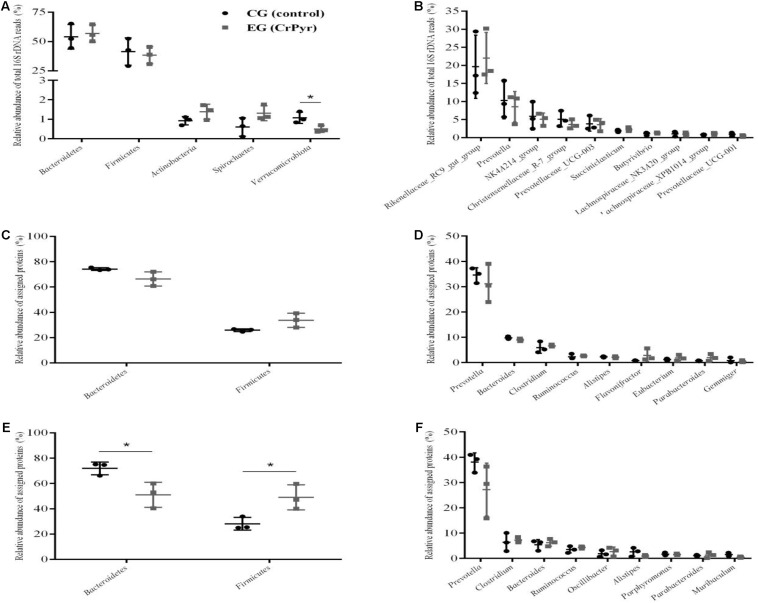
Phylogenetic classification and metaproteomics analyses of rumen fluid microbiota of beef cattle fed a CrPyr supplementation diet. **(A,B)** show phylum-level **(A)** and genus-level **(B)** rumen fluid community composition based on Illumina sequences of 16S rDNA amplicons (V3–V4 region). **(C,D)** show relative abundances of total identified proteins in rumen fluid assigned to bacterial phyla **(C)** and genera **(D)**. **(E,F)** show relative abundances of differentially expressed proteins in rumen fluid assigned to bacterial phyla **(E)** and genera **(F)**. CG, control group; EG, experimental group, diet supplemented with 60 g/day CrPyr. *n* = 3 per group. *P < 0.05.

16S rDNA sequencing and metaproteomics were combined to investigate the effects of CrPyr on both the structure and function of rumen fluid microbiota of beef cattle. 16S rDNA sequencing revealed that bacteria belonging to the phyla *Bacteroidetes* and *Firmicutes* comprised most (the average coverage was ∼95.4%) of the total bacteria in the rumen fluid microbiota of the CG and EG. The remaining bacteria were mainly members of *Actinobacteria*, *Spirochaetes*, and *Verrucomicrobiota* (Figure 4A). The 16S rDNA relative abundances (abbreviated as 16SDA hereafter) of members of *Bacteroidetes* increased from 54.08% with CG to 56.89% with EG, the *Firmicutes* 16SDA decreased from 41.47% to 38.35%, and the *Verrucomicrobiota* 16SDA was significantly different between the two groups (*P* < 0.05; Figure 4A). At the genus level, the *Rikenellaceae_RC9_gut_group* was the dominant bacteria, and the other abundant genera were *Prevotella*, *NK4A214_group*, *Christensenellaceae_R-7_group*, *Prevotellaceae_UCG-003*, and *Succiniclasticum* (Figure 4B). At the species level, the *unclassified_g_Rikenellaceae_RC9_gut_group* 16SDA was significantly higher in the EG group than in the CG group (*P* < 0.05; Supplementary Figure 2).

Metaproteomics showed that the protein relative abundances (abbreviated as PRA hereafter) of members of *Bacteroidetes* in total quantified proteins decreased from 74.15% with CG to 66.30% with EG, and the *Firmicutes* PRA increased from 25.85% to 33.70% (Figure 4C). At the genus level, the proteomic data showed that members of *Prevotella* were the dominant proteins, and the other abundant proteins were members of *Bacteroides*, *Clostridium*, *Ruminococcus*, and *Alistipes* (Figure 4D).

### Composition and Functional Classification of Differentially Expressed Proteins as Revealed by Metaproteomics

The metaproteomics data showed all 700 (373 unique in EG + 206 unique in CG + 121 differential overlapping, Supplementary Table 4). DEPs were members of *Bacteroidetes* and *Firmicutes* (Figure 4E). The *Bacteroidetes* PRA was increased, and the *Firmicutes* PRA was decreased significantly in the EG group than those in the CG group (*P* < 0.05). At the genus level, similar to the total quantified proteins (Figure 4D), the members of *Prevotella* were also the dominant proteins in DEPs (Figure 4F), and the other abundant proteins were *Clostridium*, *Bacteroides*, and *Ruminococcus* (Figure 4F).

**FIGURE 5:**
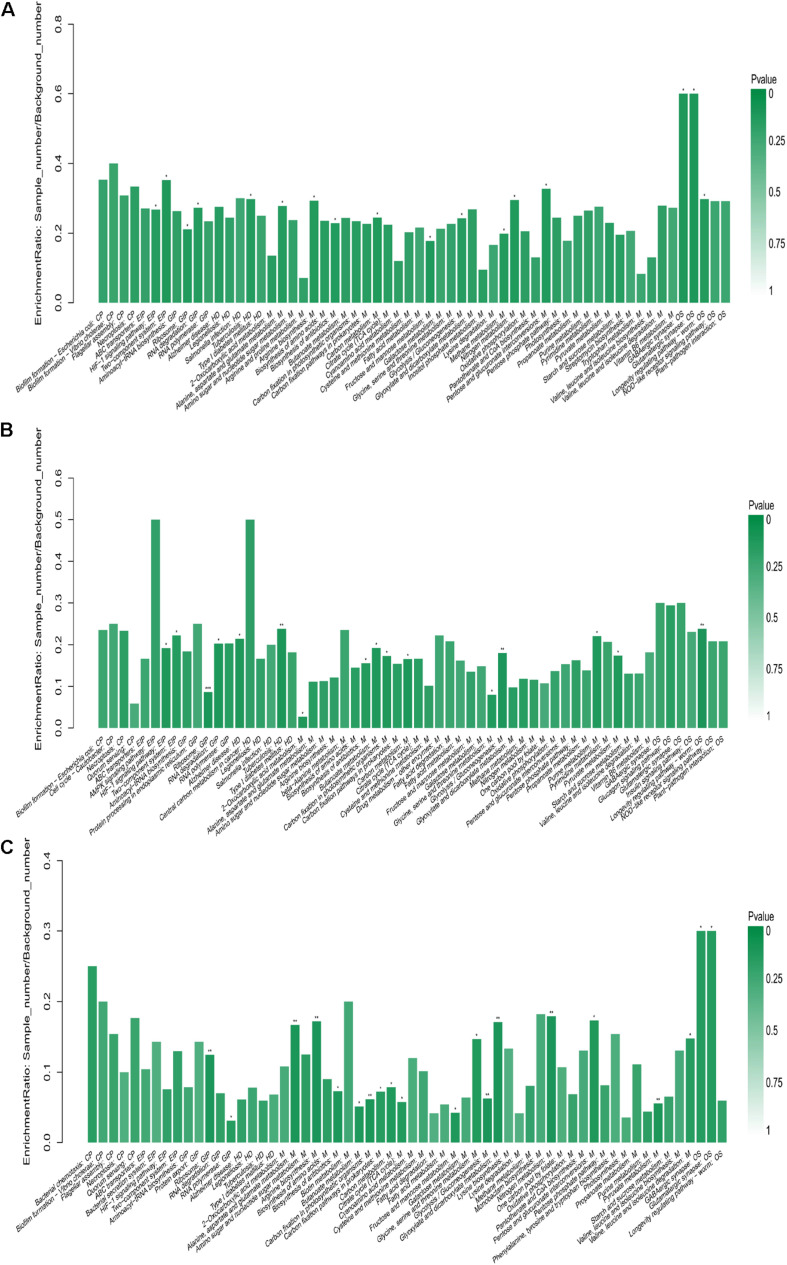
KEGG enrichment of differentially expressed proteins. **(A)** KEGG enrichment analysis of all differentially expressed proteins. **(B)** KEGG enrichment analysis of all up-regulated differentially expressed proteins (experimental group vs. control group, EG vs. CG). **(C)** KEGG enrichment analysis of all down-regulated differentially expressed proteins (experimental group vs. control group, EG vs. CG). CP, cellular processes; EIP, environmental information processing; GIP, genetic information processing; HD, human diseases; M, metabolism; and OS, organismal systems.

Kyoto Encyclopedia of Genes and Genomes (KEGG) pathway enrichment analysis was used to explore the biological pathways for DEPs between the rumen fluid microbiota of EG and CG. Figure 5A shows the KEGG pathway enrichment analysis of all DEPs. Figure 5B shows the KEGG pathway enrichment analysis of all up-regulated DEPs; diet supplemented with CrPyr significantly enriched proteins involved in environmental information processing (HIF-1 signaling pathway, two-component systems), genetic information processing (ribosome, RNA degradation), human diseases (Alzheimer’s disease, tuberculosis), metabolism (2-oxocarboxylic acid metabolism, biosynthesis of antibiotics, butanoate metabolism, carbon fixation in photosynthetic organisms, carbon metabolism, glycine, serine, and threonine metabolism, glycolysis/gluconeogenesis, purine metabolism, and pyruvate metabolism), organismal systems (longevity regulating pathway-worm). Figure 5C shows the KEGG pathway enrichment analysis of all down-regulated DEPs; diet supplemented with CrPyr significantly decreased proteins involved in genetic information processing (ribosome, RNA polymerase), metabolism (alanine, aspartate, and glutamate metabolism, arginine biosynthesis, biosynthesis of antibiotics, butanoate metabolism, carbon fixation in photosynthetic organisms, carbon fixation pathway in prokaryotes, carbon metabolism, citrate cycle, fructose and mannose metabolism, glycine, serine, and threonine metabolism, glycolysis/gluconeogenesis, glyoxylate and dicarboxylate metabolism, nitrogen metabolism, pentose and glucuronate interconversions, pyruvate metabolism, valine, leucine, and isoleucine degradation), and organismal systems (GABAergic synapse, glutamatergic synapse).

**FIGURE 6:**
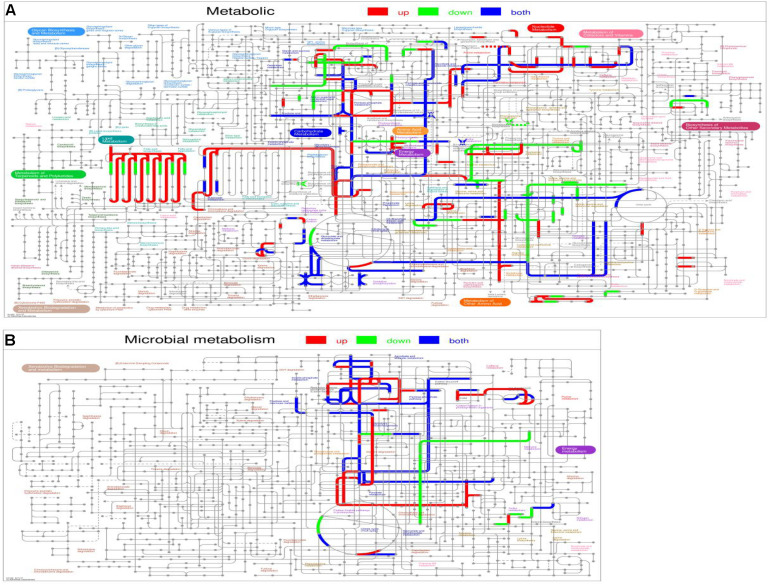
Interactive Pathways Explorer (iPath) analysis. **(A)** Metabolic. **(B)** Microbial metabolism. Red lines show up-regulated pathways, green lines show down-regulated pathways, and blue lines show both up-regulated and down-regulated pathways.

Interactive Pathways Explorer (IPath) analysis was also used to visualize the mutual relationship of DEPs in metabolic (Figure 6A) and microbial metabolism (Figure 6B), on which red lines show up-regulated pathways, green lines show down-regulated pathways, and blue lines show both up-regulated and down-regulated pathways. As shown in metabolic and microbial metabolism, the up-regulated pathways mainly including lipid metabolism, glycolysis/gluconeogenesis, and pyruvate metabolism.

### Administration of CrPyr Affected Core Enzymes Related to Key Metabolism

#### Fatty Acid Metabolism

**FIGURE 7:**
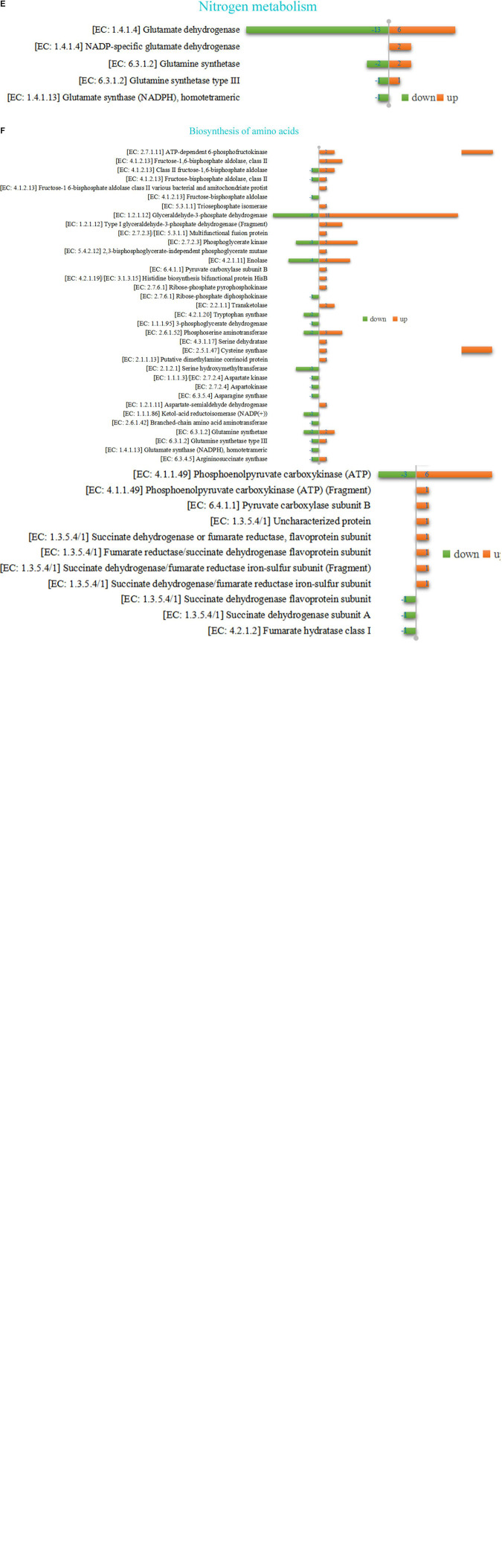
Protein identity and regulation involved in several important pathway in rumen fluid samples of beef cattle fed with a CrPyr supplementation diet (experimental group vs. control group, EG vs. CG). **(A)** Fatty acid metabolism. **(B)** Pyruvate metabolism. **(C)** Glycolysis/gluconeogenesis. **(D)** Citrate cycle (TCA cycle). **(E)** Nitrogen metabolism. **(F)** Biosynthesis of amino acids.

As shown in Figure 7A and Supplementary Table 5, diet supplemented with CrPyr up-regulated the Acyl-CoA dehydrogenase (EC: 1.3.8.1) from *Lachnoclostridium* sp., *Lachnospiraceae bacterium*, and *Oscillibacter* sp. but down-regulated this enzyme from *Muribaculum.* Exposure to CrPyr up-regulated the acetyl-CoA C-acetyltransferase (ACAA; EC: 2.3.1.9) from *Oscillibacter* sp. Moreover, the 3-oxoacyl-[acyl-carrier-protein] synthase 2 (EC: 2.3.1.179) from *Lentimicrobiaceae bacterium* was up-regulated, and the 3-oxoacyl-[acyl-carrier-protein] reductase (EC: 1.1.1.100) from *Prevotella* sp. *BP1-148* was down-regulated. The description of DEPs involved in fatty acid (FA) metabolism is shown in Supplementary Table 5.

#### Pyruvate Metabolism

As shown in Figure 7B, for the pyruvate metabolism pathway, 62 DEPs were identified in the EG compared with the CG, of which 47 proteins were up-regulated and 15 proteins were down-regulated. Of the 47 up-regulated proteins, most were pyruvate, phosphate dikinase (PPDK; EC: 2.7.9.1; LCI, 1970). Others were pyruvate:ferredoxin (flavodoxin) oxidoreductase (Sales et al., 2021) and pyruvate-flavodoxin oxidoreductase (Thomas et al., 2017; EC: 1.2.7.1), phosphoenolpyruvate carboxykinase (ATP; Dai et al., 2017; EC: 4.1.1.49), and so on. The description of DEPs involved in pyruvate metabolism is shown in Supplementary Table 6.

#### Glycolysis/Gluconeogenesis

As shown in Figure 7C, for the glycolysis/gluconeogenesis, 101 DEPs were identified in the EG compared with the CG, of which 75 proteins were up-regulated and 26 proteins were down-regulated. Except for EC: 1.2.7.1, EC: 4.1.1.49, and EC: 1.2.7.11, which were also involved in pyruvate metabolism, the most up-regulated protein was glyceraldehyde-3-phosphate dehydrogenase (Van Soest et al., 1991), including 3 Type I glyceraldehyde-3-phosphate dehydrogenase (Fragment; EC: 1.2.1.12). Others were fructose-1,6-bisphosphate aldolase, class II and its isozyme (Dai et al., 2017; EC: 4.1.2.13), phosphoglycerate kinase (Uyeno et al., 2010; EC: 2.7.2.3), pyrophosphate-fructose 6-phosphate 1-phosphotransferase (Salles et al., 2010; EC: 2.7.1.90), enolase (Salles et al., 2010; EC: 4.2.1.11), and so on. The description of DEPs involved in glycolysis/gluconeogenesis is shown in Supplementary Table 7.

#### Citrate Cycle

As shown in Figure 7D, for the citrate cycle, 39 DEPs were identified in the EG compared with CG, of which 29 proteins were up-regulated and 10 proteins were down-regulated. Except for EC: 1.2.7.1, EC: 1.2.7.11, EC: 4.1.1.49, and EC: 6.4.1.1, which are also involved in pyruvate metabolism, the most up-regulated protein was succinate dehydrogenase/fumarate reductase and its isozyme (Salles et al., 2010; EC: 1.3.5.4; EC: 1.3.5.1). The description of DEPs involved in citrate cycle is shown in Supplementary Table 8.

### Nitrogen Metabolism and Biosynthesis of Amino Acids

As shown in Figure 7E, for the nitrogen metabolism pathway, 28 DEPs were identified in EG compared with CG, of which 11 proteins were up-regulated and 17 proteins were down-regulated. Of the 11 up-regulated proteins, eight were glutamate dehydrogenase (GDH; EC: 1.4.1.4) and three glutamine synthetase (GS; EC: 6.3.1.2). As shown in Figure 7F, for the biosynthesis of amino acids, 94 DEPs were identified in the EG compared with the CG, of which 58 proteins were up-regulated and 36 proteins were down-regulated. The description of DEPs involved in nitrogen metabolism and biosynthesis of amino acids is shown in Supplementary Tables 9, 10.

## Discussion

The LWSI classifications for heat stress are as follows: normal, ≤74; alert, 74 ≤ THI ≤ 79; danger, 79 ≤ THI ≤ 84; and emergency, THI ≥ 84 (LCI, 1970). The present study indicated that the experimental cattle were in a state of high heat stress due to the high temperature and humidity during the summer months. Consequently, to seek thermoregulation, the animal employs a series of physiological and metabolic changes, including an increased rectal temperature, respiration rate, panting and open-mouth breathing, water consumption, and a decrease in feed intake (Marai et al., 2007). Body temperature is an excellent indicator of an animal’s susceptibility to heat load, and the body core temperature of cattle in thermo-neutral conditions is maintained between 38 and 39.2°C (Ammer et al., 2016). In this study, in comparison to the control group, dietary supplementation with CrPyr significantly decreased the rectal temperature of cattle, which means CrPyr could relieve a heat stress-induced higher body temperature. Creatine is an osmotically active substance, and the osmotic effect of creatine might affect the thermoregulatory processes during exercise. Some research has suggested that, in humans, a potential mechanism involving increased extracellular fluid induced by added creatine may help the body deal with heat stress (Mendel et al., 2005).

Rumen pH value and rumen bacterial populations are decreased during heat stress (Tajima et al., 2007; Bernabucci et al., 2009). Rumen pH value is the result of the combined effect of factors such as the VFA in rumen fluid, buffer salt in saliva, organic acid produced by metabolism, NH_3_-N level, and emptying speed of rumen fluid. The production of NH_3_-N in the rumen can increase the rumen pH value. In the present study, CrPyr supplementation increased the rumen pH value. This might be attributed to the slow continuous release of ammonia from creatine. As previously reported, calcium pyruvate supplementation of goat diets could increase the content of VFA in the rumen (Chen et al., 2006; Ran et al., 2008). Moreover, creatine has been widely used as a nitrogen supplement in ruminant nutrition in the past few decades (McLaren, 1964). However, there was no significant difference in VFA or NH_3_-N concentration for beef cattle fed CrPyr in heat stress. This may be due to that the VFA production induced by pyruvate and the simultaneously slow continuous release of ammonia from creatine in the rumen were partly used by rumen microbes to synthesize proteins. Consistently, we found CrPyr increased the content of MCP in the present study.

In this study, CrPyr supplementation significantly increased the proportion of *unclassified_g_Rikenellaceae_RC9_gut_group* in the rumen of heat-stressed beef cattle (Supplementary Figure 2), which can produce VFA (mainly acetate and propionate) and promote cellulose digestion. Also, Cheng (2019) reported that rumen calcium pyruvate perfusion based on a high-concentrate diet (F:C, 6:4) increased the ratio of *Rikenellaceae_RC9_gut_group* in the rumen of nonpregnant dairy goats. This may illustrate that pyruvate is favorable for *Rikenellaceae_RC9_gut_group* reproduction and further contributes benefits to cellulose digestion. While our results showed that the neutral detergent fiber and acid detergent fiber digestibility between the EG and CG groups was not significantly different, this may be due to the main ruminal cellulolytic bacteria including *Ruminococcus albus*, *Ruminococcus flavefaciens*, and *Fibrobacter succinogenes* were not affected by CrPyr.

The results in the present study showed that supplementing with CrPyr significantly increased the crude fat digestibility. Simultaneously, the IPath analysis showed the administration of CrPyr-enriched proteins involved in lipid metabolism (Figure 6A), including those involved in FA degradation and FA biosynthesis, in the rumen fluid microbiota of beef cattle. The present study was the first metabolic evidence to demonstrate that CrPyr promotes the expression of enzymes involved in lipid metabolism of the rumen fluid microbiota of beef cattle. Indeed, in the rumen, bacteria are largely responsible for biohydrogenation of dietary unsaturated FA (Harfoot and Hazlewood, 1997), and few studies in the literature have reported rumen bacteria FA β-oxidation (Emmanuel, 1978). However, in the anaerobic digestion process long-chain FAs and short-chain FAs can be degraded *via* the β-oxidation pathway catalyzed by bacteria (Kim et al., 2004; Hao et al., 2020), which may suggest that FA β-oxidation may also occur under anaerobic conditions in the rumen. The metaproteomics screening in the present study detected that the expressions of short-chain acyl-CoA dehydrogenase (SCAD) and ACAA (Supplementary Figure 3 and Supplementary Table 5) were significantly up-regulated in the CrPyr-supplemented group compared with the control group. Acyl-CoA dehydrogenase catalyzes the initial rate-limiting step, and ACAA catalyzes the last step of the FA β-oxidation. They are all key enzymes of the FA oxidation (Andresen et al., 1999; Zhang et al., 2018). In the present study, the up-regulation of SCAD from *Lachnoclostridium* sp., *L. bacterium*, and *Oscillibacter* sp. catalyzes the dehydrogenation of hexanoyl-CoA and butanoyl-CoA. The up-regulation of ACAA from *Oscillibacter* sp. catalyzes the reaction of 3-oxo-hexanoyl-CoA and CoA to form acetyl-CoA and butanoyl-CoA, and the formation of two molecules of acetyl-CoA from acetoacetyl-CoA. Then, acetyl-CoA enters into the citrate cycle providing energy for metabolism. Moreover, Zhu et al. (2017) reported that the dominant species in the microbiota composition were *L. bacterium 10_1* and *Oscillibacter* sp. *1_3* in stool samples of mice with resistance to high-fat diet-induced obesity. Ma et al. (2020) showed that *L. bacterium DW67* was one of the key mediators in exerting antiobesity induced by cold-water brewed green tea. This may suggest that *L. bacterium* and *Oscillibacter* sp. have potential for FA oxidation in rumen. The above SCAD and ACAA catalyzed reactions could promote FA degradation, which may explain the increased digestibility of crude fat in the CrPyr-supplemented group. On the other hand, the ATP generation during FA β-oxidation may help decrease oxidative stress, regulate energy metabolism, and further improve the rumen fermentation characteristic under heat stress.

Pyruvate is the intermediate product of carbohydrate fermentation by rumen microorganisms (Nagaraja, 2016). Our metaproteomics analyses showed that the administration of CrPyr significantly increased the expression of enzymes involved in pyruvate metabolism, glycolysis/gluconeogenesis, and citrate cycle pathway. Phosphoenolpyruvate (PEP)/pyruvate interconversion is a major metabolic point in glycolysis/gluconeogenesis (Chen et al., 2019). In the case of the pyruvate-to-PEP conversion, the reaction proceeds through diversified metabolic reactions. For instance, in propionic acid bacteria, PPDK is the primary enzyme that directly converts pyruvate to PEP in one step (Evans and Wood, 1971). However, in most mammals, plants, and microorganisms, the reaction proceeds by two steps, catalyzed by pyruvate carboxylase (converts pyruvate to oxaloacetate) and PEP carboxykinase (interconverts oxaloacetate and PEP; Bräsen et al., 2014). In this study, the pyruvate-to-PEP conversion was promoted, proving that the PPDK was significantly enriched (including 17 up-regulated PPDK and 7 down-regulated PPDK from different bacteria). Besides, although the pyruvate carboxylase subunit B and the PEP carboxykinase (ATP) were up-regulated, this may not be the pyruvate-to-PEP conversion proceeded by two steps, since in some anaerobic bacteria, PEP carboxykinase carboxylates PEP to oxaloacetate with the conservation of energy as ATP (Zhang et al., 2009; Asanuma et al., 2010). The above up-expressed enzymes involved in pyruvate metabolism means that CrPyr supplementation promoted the conversions of pyruvate to PEP, pyruvate to PEP to oxaloacetate, and pyruvate to oxaloacetate in rumen bacteria.

Moreover, in pyruvate metabolism, we also found that the enzymes involved in the conversion of pyruvate to acetyl-CoA or (S)-malate were promoted in the CrPyr administration group. In several microorganisms, pyruvate-flavodoxin oxidoreductase is responsible for acetyl-CoA formation from pyruvate in a single step (Ferrer et al., 2007). Our metaproteomics analysis showed that the up-regulated enzymes involved in the pyruvate-to-acetyl-CoA conversion include 13 pyruvate-flavodoxin oxidoreductase and its isozymes from different bacteria, 2-oxoglutarate ferredoxin oxidoreductase subunit alpha from *Prevotella* sp. *khp7*, and 2-oxoglutarate ferredoxin oxidoreductase subunit beta from *Prevotella* sp. *tf2-5*. Generally, microbes have five metabolic pathways involved in malate biosynthesis. Two of these pathways take pyruvate as the metabolic starting point: (I) pyruvate is the first to convert to oxaloacetate *via* the pyruvate carboxylase, and then malate dehydrogenase converts oxaloacetate to malate; (II) pyruvate is directly converted to malate *via* malic enzyme in one step (Zhou et al., 2015). In the present study, CrPyr administration up-regulated the malate dehydrogenase from *Bacteroides xylanolyticus*, NAD-dependent malic enzyme from *Clostridium homopropionicum DSM 5847*, and allosteric NADP-dependent malic enzyme from *Prevotella ruminicola*. The above up-expressed enzymes involved in pyruvate metabolism means that CrPyr supplementation promoted the conversions of pyruvate to acetyl-CoA, pyruvate to malate, and pyruvate to oxaloacetate to malate in rumen bacteria.

In bacteria, external glucose is transported into the cells and phosphorylated by the PEP:sugar phosphotransferase system (PTS). PEP as the phosphoryl donor to phosphorylate sugars is the primary source of energy for glucose uptake and phosphorylation (Zhang et al., 2009; Erni, 2013). In the present study, CrPyr supplementation increased the expression of enzymes involved in the conversion of pyruvate to PEP, which may increase the pool of PEP available for PTS and to facilitate glucose phosphorylation transport in bacteria, thereby up-regulating glycolysis. Consistently, in this study, the enzymes involved in glycolysis/gluconeogenesis were up-regulated, including 2,3-bisphosphoglycerate-independent phosphoglycerate mutase, phosphoglycerate kinase, glyceraldehyde-3-phosphate dehydrogenase, fructose-1,6-bisphosphate aldolase, pyrophosphate-fructose 6-phosphate 1-phosphotransferase, glucose-6-phosphate isomerase, phosphoglucomutase, and aldose 1-epimerase. Of those enzymes, the glyceraldehyde-3-phosphate dehydrogenase was most enriched, with 21 up-regulated and 6 down-regulated proteins from different bacteria.

Glycolysis and the citrate cycle provide sources of energy within the rumen (Czerkawski, 1986). Recently, one study using a metagenomics approach elucidated that the citrate cycle (Krebs) exists in rumen microbiota metabolism (Mu et al., 2021). In this study, the acetyl-CoA generated from FA β-oxidation and the oxaloacetate, malate, and acetyl-CoA generated from pyruvate metabolism could enter the citrate cycle, promote the citrate cycle metabolism, and provide energy. Consistent with this, we found that 29 proteins were up-regulated and 10 proteins were down-regulated in the citrate cycle pathway. The up-regulation of enzymes involved in citrate cycle metabolism might help to produce more α-ketoglutarate. When ammonia and α-ketoglutarate are in a certain ratio, the efficiency of MCP synthesis can be optimized without causing the accumulation of ammonia or α-ketoglutarate. This is rumen energy-nitrogen equilibrium (Feng and Lu, 2007). In rumen, ammonium is the preferred nitrogen source for microbial growth. Creatine, as an important nitrogen-containing compound in protein and energy metabolism, has been widely used as a nitrogen supplement in ruminant nutrition due to its slow continuous release of ammonia (McLaren, 1964; Navrátil et al., 2009). The results from nitrogen metabolism indicated that CrPyr administration led to 8 up-regulated bacterial reduced NADPH-specific GDH (EC: 1.4.1.4) and 13 down-regulated bacterial NADPH-specific GDH. We also found three up-regulated and three down-regulated bacterial GS (EC: 6.3.1.2) and one down-regulated bacterial glutamate synthase (GOGAT; EC: 1.4.1.13). The GDH and GS-GOGAT pathways are two classic routes for ammonia assimilation in bacteria (Wang and Tan, 2013). In the GDH pathway, the NADH-specific GDH serves to degrade glutamate:

(1) $$\begin{array}{c} \text{glutamate}+{\text{NAD}}^{\underset{\to }{\mathrm{NADH}\text{-}\mathrm{GDH}}}\alpha \text{-}\mathrm{ketoglutarate}\\ \;+\mathrm{NADH}+{\mathrm{NH}}_{3} \end{array}$$

while NADPH-specific GDH serves for glutamate synthesis:

(2) $$\begin{array}{c} \alpha \text{-}\mathrm{ketoglutarate}+\mathrm{NADPH}+{\mathrm{NH}}_{3}^{\underset{\to }{\mathrm{NADPH}\text{-}GDH}}\text{NADP}\\ \;+\mathrm{glutamate} \end{array}$$

Phibbs and Bernlohr (1971) in the GS-GOGAT pathway, GS is a ubiquitous enzyme that catalyzes the ATP-dependent amidation glutamate to generate glutamine; GOGAT catalyzes the reductive transfer of the amide group of glutamate. In this study, the GDH pathway appears to be the predominant route of ammonia assimilation. We further analyzed the DEPs involved in the biosynthesis of amino acids, of which 58 proteins were up-regulated and 36 proteins were down-regulated; these results showed that CrPyr was helpful to amino acid biosynthesis of bacteria in rumen fluid. Moreover, since this project assumed only the *in vivo* model, pure bacterial lines whose enzymatic abundance were changed between CrPyr-supplemented and -unsupplemented treatments were not isolated and assessed *in vitro*. These experiments need to further improve in the further study.

## Conclusion

**FIGURE 8:**
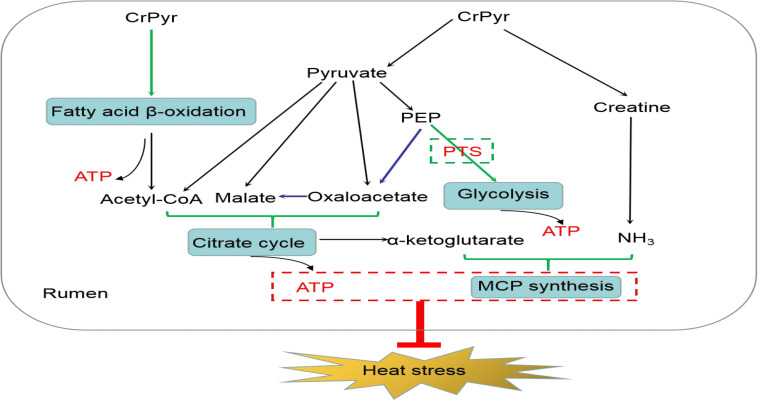
Proposed model of creatine pyruvate relieving heat stress in rumen of beef cattle.

In conclusion, CrPyr administration increased the digestibility of crude fat through promoting the expression of enzymes involved in FA β-oxidation of rumen bacteria and up-regulated the expression of enzymes involved in glycolysis/gluconeogenesis through increasing the pool of PEP available for PTS and to facilitate glucose phosphorylation transport. The acetyl-CoA generated from FA β-oxidation and the oxaloacetate, malate, and acetyl-CoA generated from pyruvate metabolism promote the citrate cycle metabolism and might help to produce more α-ketoglutarate. α-Ketoglutarate, together with the NH_3_ released by creatine, promoted the synthesis of MCP in the rumen. The increased production of ATP and the up-regulation synthesis of MCP in rumen may help decrease oxidative stress, regulate energy metabolism, and further improve the rumen fermentation characteristic of beef cattle under heat stress (Figure 8).

## Data Availability Statement

The datasets presented in this study can be found in online repositories. The names of the repository/repositories and accession number(s) can be found in the article/Supplementary Material.

## Ethics Statement

The animal study was reviewed and approved by the Committee for the Care and Use of Experimental Animals at Jiangxi Agricultural University (JXAULL-20190017).

## Author Contributions

YL and MQ designed the overall study. YL, YZ, XZ, LL, QQ, and KO performed the experiments. YL wrote the manuscript. All the authors contributed to the article and approved the submitted version.

## Conflict of Interest

The authors declare that the research was conducted in the absence of any commercial or financial relationships that could be construed as a potential conflict of interest.

## Publisher’s Note

All claims expressed in this article are solely those of the authors and do not necessarily represent those of their affiliated organizations, or those of the publisher, the editors and the reviewers. Any product that may be evaluated in this article, or claim that may be made by its manufacturer, is not guaranteed or endorsed by the publisher.

## Footnotes

1
http://rdp.cme.msu.edu/

2
http://proteomecentral.proteomexchange.org
