# Supplementary figures and images for: Dietary Supplementation With Creatine Pyruvate Alters Rumen Microbiota Protein Function in Heat-Stressed Beef Cattle
Source: Front Microbiol. 2021 Aug 27;12:715088. doi: 10.3389/fmicb.2021.715088 (PMC8431830; doi:10.3389/fmicb.2021.715088)

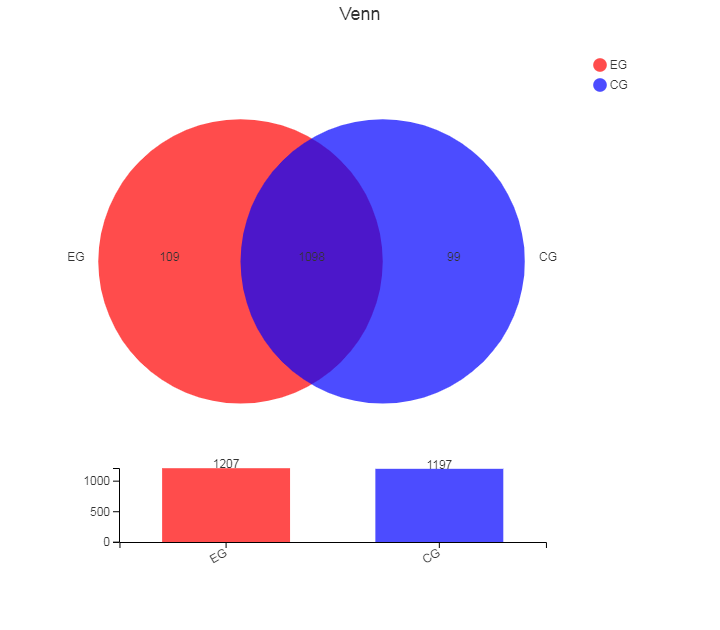

Supplement: Supplementary file 1 [file Image_1.TIF]

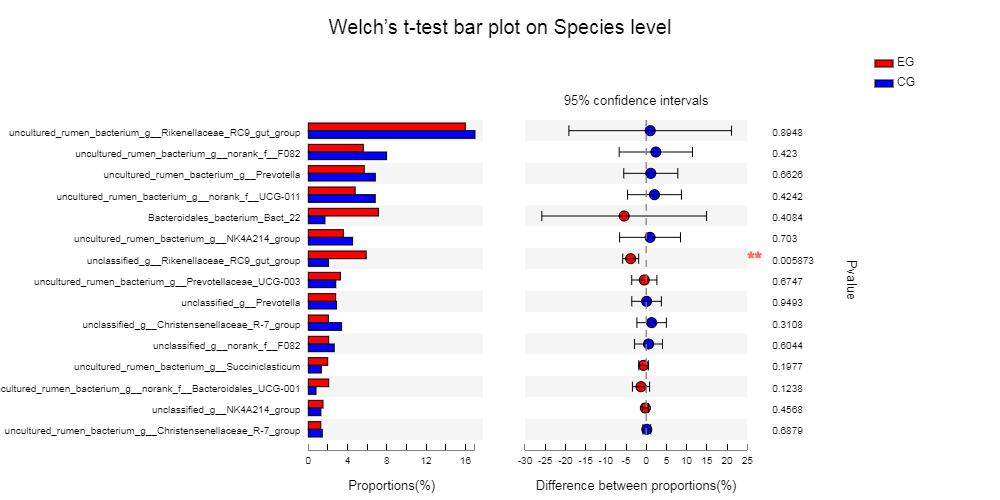

Supplement: Supplementary file 2 [file Image_2.TIF]

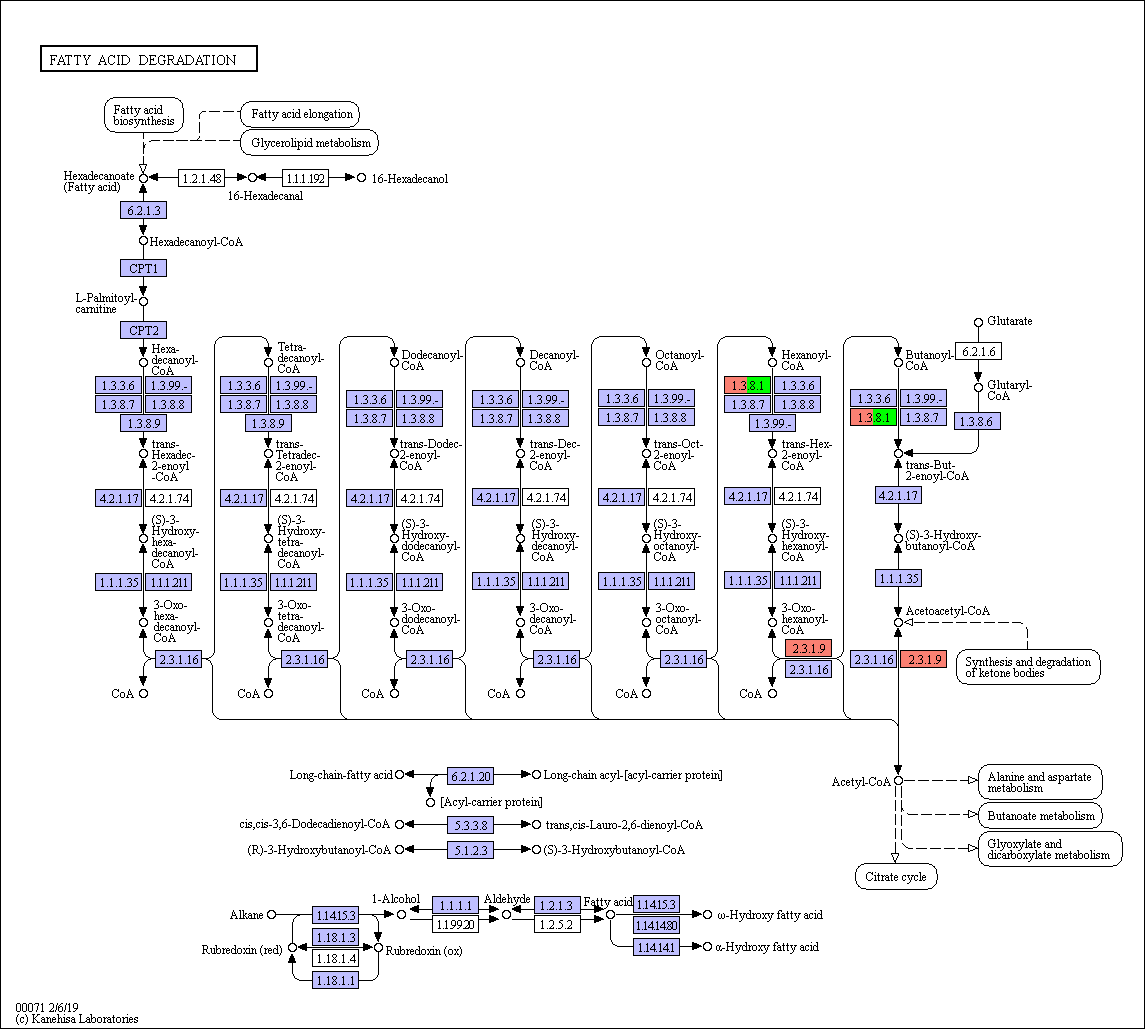

Supplement: Supplementary file 3 [file Image_3.TIF]
