# Supplementary material for: Dietary Supplementation With Creatine Pyruvate Alters Rumen Microbiota Protein Function in Heat-Stressed Beef Cattle
Source: Front Microbiol. 2021 Aug 27;12:715088. doi: 10.3389/fmicb.2021.715088 (PMC8431830; doi:10.3389/fmicb.2021.715088)
Supplement: Supplementary file 4 [file Table_1.DOC]

**Table S1. Composition and nutrient levels of the experimental diet (air-dry basis, %)**

| Ingredients | Content | Nutrient levels | Content |
| --- | --- | --- | --- |
| Peanut vine | 40.00 | Dry matter | 87.20 |
| Corn | 30.70 | Crude protein | 10.98 |
| Wheat bran | 25.40 | Crude fat | 3.48 |
| Soybean meal | 2.00 | Ash | 9.60 |
| Sodium bicarbonate | 0.20 | Neutral detergent fiber | 45.03 |
| Salt | 0.10 | Acid detergent fiber | 22.44 |
| 4% Premix^1^ | 1.60 |  |  |

^1^The premix is provided per kilogram of diet: 3200 mg of iron as iron sulfate, 1500 mg of manganese as manganous oxide, 2000 mg of zinc as zinc oxide, 650 mg of copper as copper sulfate, 35 mg of iodate as calcium iodate, 10 mg of selenium as sodium selenite, 10 mg of cobalt as cobalt chloride, 130 g of calcium as calcium carbonate, 30 g of phosphorus as calcium hydrogen phosphate, 45 mg retinyl acetate, 40 μg cholecalciferol, and 3.0 mg DL-α-tocopheryl acetate.
