# Supplementary material for: Dietary Supplementation With Creatine Pyruvate Alters Rumen Microbiota Protein Function in Heat-Stressed Beef Cattle
Source: Front Microbiol. 2021 Aug 27;12:715088. doi: 10.3389/fmicb.2021.715088 (PMC8431830; doi:10.3389/fmicb.2021.715088)
Supplement: Supplementary file 5 [file Table_2.DOC]

**Table S2.** Effect of creatine pyruvate on rumen microbial diversity indices

| Item | CG^1^ | EG^2^ | *P-*value |
| --- | --- | --- | --- |
| Ace | 1040.52 ± 25.16 | 1100.06 ± 48.36 | 0.237 |
| Chao 1 | 1048.06 ± 20.33 | 1097.03 ± 52.25 | 0.105 |
| Shannon | 5.11 ± 0.13 | 5.13 ± 0.24 | 0.382 |
| Simpson | 0.020 ± 0.00 | 0.024 ± 0.01 | 0.105 |
| Coverage | 0.99 ± 0.00 | 0.99 ± 0.00 | 0.115 |

^1^CG = control group.

^2^EG = experimental group, diet supplemented with 60 g/d CrPyr.
