# Supplementary material for: Dietary Supplementation With Creatine Pyruvate Alters Rumen Microbiota Protein Function in Heat-Stressed Beef Cattle
Source: Front Microbiol. 2021 Aug 27;12:715088. doi: 10.3389/fmicb.2021.715088 (PMC8431830; doi:10.3389/fmicb.2021.715088)
Supplement: Supplementary file 8 [file Table_5.DOC]

**Table S5.** Protein identity and regulation involved in fatty acid metabolism pathway in rumen fluid samples of beef cattle fed with a CrPyr supplementation diet

| EC number | Regulate | Accession | Description |
| --- | --- | --- | --- |
| EC: 1.3.8.1 | up | A0A3B8U9Q8 | Acyl-CoA dehydrogenase OS=Lachnospiraceae bacterium OX=1898203 GN=DCF49_07485 PE=3 SV=1 |
|  |  | A0A350TKZ0 | Acyl-CoA dehydrogenase OS=Oscillibacter sp. OX=1945593 GN=DCX96_06760 PE=3 SV=1 |
|  |  | A0A3B9K919 | Acyl-CoA dehydrogenase OS=Lachnoclostridium sp. OX=2028282 GN=DCG70_08750 PE=3 SV=1 |
|  |  | K0Y482 | Rubredoxin-like domain-containing protein OS=Lachnoanaerobaculum sp. OBRC5-5 OX=936595 GN=HMPREF1135_00569 PE=3 SV=1 |
|  | down | A0A1Y4CDN8 | Acyl-CoA dehydrogenase OS=Muribaculum sp. An287 OX=1965623 GN=B5F81_00050 PE=3 SV=1 |
| EC: 2.3.1.9 | up | A0A352RP61 | Acetyl-CoA C-acetyltransferase OS=Oscillibacter sp. OX=1945593 GN=DC027_06405 PE=3 SV=1 |
| EC: 2.3.1.179 | up | A0A2E2XYI4 | 3-oxoacyl-[acyl-carrier-protein] synthase 2 OS=Lentimicrobiaceae bacterium OX=2026756 GN=fabF PE=3 SV=1 |
| EC: 1.1.1.100 | down | A0A1G7SH33 | 3-oxoacyl-[acyl-carrier-protein] reductase OS=Prevotella sp. BP1-148 OX=645274 GN=SAMN04487901_101313 PE=3 SV=1 |
