# Supplementary material for: Dietary Supplementation With Creatine Pyruvate Alters Rumen Microbiota Protein Function in Heat-Stressed Beef Cattle
Source: Front Microbiol. 2021 Aug 27;12:715088. doi: 10.3389/fmicb.2021.715088 (PMC8431830; doi:10.3389/fmicb.2021.715088)
Supplement: Supplementary file 9 [file Table_6.DOC]

**Table S6.** Protein identity and regulation involved in pyruvate metabolism pathway in rumen fluid samples of beef cattle fed with a CrPyr supplementation diet

| EC number | Regulate | Accession | Description |
| --- | --- | --- | --- |
| EC: 2.7.9.1 | up | A0A416GBK6 | Pyruvate, phosphate dikinase OS=Bacteroides sp. OF04-15BH OX=2292281 GN=DXA74_09435 PE=3 SV=1 |
|  |  | A0A1Y4RRZ4 | Pyruvate, phosphate dikinase OS=Lachnoclostridium sp. An14 OX=1965562 GN=B5E84_06395 PE=3 SV=1 |
|  |  | A0A143ZVA2 | Pyruvate, phosphate dikinase OS=Eubacteriaceae bacterium CHKCI005 OX=1780381 GN=ppdK PE=3 SV=1 |
|  |  | A0A0K9N9L3 | Pyruvate, phosphate dikinase OS=Dorea sp. D27 OX=658665 GN=HMPREF0980_03260 PE=3 SV=1 |
|  |  | A0A162NEF4 | Pyruvate, phosphate dikinase OS=Clostridium coskatii OX=1705578 GN=ppdK PE=3 SV=1 |
|  |  | A0A1Y4ISV6 | Pyruvate, phosphate dikinase OS=Lachnoclostridium sp. An196 OX=1965583 GN=B5F29_02230 PE=3 SV=1 |
|  |  | R7GHK4 | Pyruvate, phosphate dikinase OS=Clostridium sp. CAG:307 OX=1262795 GN=BN598_00612 PE=3 SV=1 |
|  |  | A0A359CV26 | Pyruvate, phosphate dikinase OS=Bacteroidales bacterium OX=2030927 GN=DEH02_03385 PE=3 SV=1 |
|  |  | R6CWG8 | Pyruvate, phosphate dikinase OS=Clostridium sp. CAG:594 OX=1262826 GN=BN726_00314 PE=3 SV=1 |
|  |  | R6NKT7 | Pyruvate, phosphate dikinase OS=Clostridium sp. CAG:413 OX=1262803 GN=BN649_00247 PE=3 SV=1 |
|  |  | A0A1K1WCZ3 | Pyruvate, phosphate dikinase OS=Ruminococcus flavefaciens OX=1265 GN=SAMN04487832_104212 PE=3 SV=1 |
|  |  | A0A3D4EAU5 | Pyruvate, phosphate dikinase OS=Rikenellaceae bacterium OX=2049048 GN=DIT75_05190 PE=3 SV=1 |
|  |  | A0A1M4XGY0 | Pyruvate, phosphate dikinase OS=Alkalibacter saccharofermentans DSM 14828 OX=1120975 GN=SAMN02746064_01518 PE=3 SV=1 |
|  |  | A0A1I6ZFR7 | Pyruvate, phosphate dikinase OS=Lachnospiraceae bacterium XBD2001 OX=1520820 GN=SAMN02910301_2153 PE=3 SV=1 |
|  |  | A0A2T0AUG6 | Pyruvate, phosphate dikinase OS=Clostridium thermopalmarium DSM 5974 OX=1121340 GN=ppdK PE=3 SV=1 |
|  |  | F4XAC4 | Pyruvate, phosphate dikinase OS=Ruminococcaceae bacterium D16 OX=552398 GN=HMPREF0866_00242 PE=3 SV=1 |
|  |  | A0A096B0S3 | Pyruvate, phosphate dikinase OS=Prevotella buccalis DNF00853 OX=1401074 GN=HMPREF2137_02090 PE=3 SV=1 |
|  | down | A0A0F3FM62 | Pyruvate, phosphate dikinase OS=Clostridium baratii OX=1561 GN=UC77_13100 PE=3 SV=1 |
|  |  | A0A1I2JGA3 | Pyruvate, phosphate dikinase OS=Clostridium cadaveris OX=1529 GN=DBY38_12865 PE=3 SV=1 |
|  |  | A0A358PEE6 | Pyruvate, phosphate dikinase OS=Clostridiales bacterium OX=1898207 GN=DEF33_00160 PE=3 SV=1 |
|  |  | A0A1K1NR31 | Pyruvate, phosphate dikinase OS=Ruminococcus flavefaciens OX=1265 GN=SAMN02910280_2186 PE=3 SV=1 |
|  |  | E1KSV4 | Pyruvate, phosphate dikinase OS=Prevotella disiens FB035-09AN OX=866771 GN=ppdK PE=3 SV=1 |
|  |  | A0A3D5ZMS0 | Pyruvate, phosphate dikinase OS=Clostridiales bacterium OX=1898207 GN=DHU79_05840 PE=3 SV=1 |
|  |  | A0A377FNX2 | Pyruvate, phosphate dikinase OS=[Eubacterium] infirmum OX=56774 GN=ppdK PE=3 SV=1 |
| EC: 4.1.1.49 | up | A0A1I3ZPE6 | Phosphoenolpyruvate carboxykinase (ATP) OS=Lachnospiraceae bacterium KH1T2 OX=1855374 GN=pckA PE=3 SV=1 |
|  |  | A0A255SS06 | Phosphoenolpyruvate carboxykinase (ATP) OS=Prevotella bryantii OX=77095 GN=pckA PE=3 SV=1 |
|  |  | A0A415MJA9 | Phosphoenolpyruvate carboxykinase (ATP) OS=Parabacteroides distasonis OX=823 GN=pckA PE=3 SV=1 |
|  |  | A0A239QZC1 | Phosphoenolpyruvate carboxykinase (ATP) OS=Prevotellaceae bacterium MN60 OX=1945887 GN=pckA PE=3 SV=1 |
|  |  | A0A1I5HGW5 | Phosphoenolpyruvate carboxykinase (ATP) OS=Prevotella sp. tf2-5 OX=1761889 GN=pckA PE=3 SV=1 |
|  |  | A0A355XQD4 | Phosphoenolpyruvate carboxykinase (ATP) (Fragment) OS=Parabacteroides distasonis OX=823 GN=pckA PE=3 SV=1 |
|  |  | A0A1H7IVN8 | Phosphoenolpyruvate carboxykinase (ATP) OS=Pseudobutyrivibrio ruminis OX=46206 GN=pckA PE=3 SV=1 |
|  | down | A0A355VYP5 | Phosphoenolpyruvate carboxykinase (ATP) OS=Lachnospiraceae bacterium OX=1898203 GN=pckA PE=3 SV=1 |
|  |  | A0A432LL30 | Phosphoenolpyruvate carboxykinase (ATP) OS=Prevotella sp. KCOM 3155 OX=2490854 GN=pckA PE=3 SV=1 |
|  |  | A0A316N6J8 | Phosphoenolpyruvate carboxykinase (ATP) OS=Clostridiaceae bacterium OX=1898204 GN=pckA PE=3 SV=1 |
| EC: 6.4.1.1 | up | A0A1M6T8D3 | Pyruvate carboxylase subunit B OS=Prevotella ruminicola OX=839 GN=SAMN05216463_10566 PE=4 SV=1 |
| EC: 1.2.7.1 | up | A0A415ZEP4 | Pyruvate:ferredoxin (Flavodoxin) oxidoreductase OS=Butyricicoccus sp. AM05-1 OX=2292004 GN=nifJ PE=3 SV=1 |
|  |  | R6P8M9 | Pyruvate-flavodoxin oxidoreductase OS=Eubacterium sp. CAG:274 OX=1262888 GN=BN582_00957 PE=3 SV=1 |
|  |  | A0A1F8V698 | Pyruvate:ferredoxin (Flavodoxin) oxidoreductase OS=Clostridiales bacterium GWF2_38_85 OX=1797683 GN=A2Y17_09250 PE=3 SV=1 |
|  |  | A0A1Y4WGD6 | Pyruvate:ferredoxin (Flavodoxin) oxidoreductase OS=Flavonifractor sp. An100 OX=1965538 GN=B5E43_03235 PE=3 SV=1 |
|  |  | A0A166TFP4 | Pyruvate-flavodoxin oxidoreductase OS=Clostridium coskatii OX=1705578 GN=nifJ_2 PE=3 SV=1 |
|  |  | A0A1K2BLI4 | Pyruvate-ferredoxin/flavodoxin oxidoreductase OS=Ruminococcus flavefaciens OX=1265 GN=SAMN04487832_1192 PE=3 SV=1 |
|  |  | A0A353YZZ4 | Pyruvate:ferredoxin (Flavodoxin) oxidoreductase (Fragment) OS=Bacteroidales bacterium OX=2030927 GN=nifJ PE=4 SV=1 |
|  |  | A0A4D7APF0 | Pyruvate:ferredoxin (Flavodoxin) oxidoreductase OS=Dysosmobacter welbionis OX=2093857 GN=nifJ PE=3 SV=1 |
|  |  | A0A3D4RN83 | Pyruvate:ferredoxin (Flavodoxin) oxidoreductase (Fragment) OS=Bacteroidales bacterium OX=2030927 GN=nifJ PE=4 SV=1 |
|  |  | T0N108 | Pyruvate-flavodoxin oxidoreductase OS=Clostridium sp. BL8 OX=1354301 GN=M918_03780 PE=3 SV=1 |
|  |  | A0A1C5XP50 | Pyruvate synthase subunit porA OS=uncultured Flavonifractor sp. OX=1193534 GN=porA_1 PE=3 SV=1 |
|  |  | A0A1Q6SJ12 | Pyruvate:ferredoxin (Flavodoxin) oxidoreductase OS=Roseburia intestinalis OX=166486 GN=BHW46_03145 PE=3 SV=1 |
|  |  | A0A2N6AJV8 | Pyruvate:ferredoxin (Flavodoxin) oxidoreductase OS=Clostridiales bacterium OX=1898207 GN=nifJ PE=3 SV=1 |
|  | down | A0A143XWN4 | Pyruvate-flavodoxin oxidoreductase OS=Eubacteriaceae bacterium CHKCI004 OX=1780380 GN=nifJ PE=3 SV=1 |
|  |  | A0A3A9BCX9 | Pyruvate:ferredoxin (Flavodoxin) oxidoreductase (Fragment) OS=Bacteroides caecimuris OX=1796613 GN=D7W50_10630 PE=4 SV=1 |
|  |  | A0A5B7THW7 | Pyruvate:ferredoxin (Flavodoxin) oxidoreductase OS=Caloramator sp. E03 OX=2576307 GN=nifJ PE=3 SV=1 |
|  |  | A0A0K8J7N6 | Pyruvate-flavodoxin oxidoreductase OS=Herbinix luporum OX=1679721 GN=nifJ2 PE=3 SV=1 |
| EC: 1.2.7.11 | up | E6SNS3 | Pyruvate flavodoxin/ferredoxin oxidoreductase domain protein OS=Bacteroides helcogenes (strain ATCC 35417 / DSM 20613 / JCM 6297 / P 36-108) OX=693979 GN=Bache_2871 PE=4 SV=1 |
|  | up | A0A1I0PQC5 | 2-oxoglutarate ferredoxin oxidoreductase subunit alpha OS=Prevotella sp. khp7 OX=1761885 GN=SAMN04487827_1930 PE=4 SV=1 |
|  | up | A0A1I5HE68 | 2-oxoglutarate ferredoxin oxidoreductase subunit beta OS=Prevotella sp. tf2-5 OX=1761889 GN=SAMN04487852_101322 PE=4 SV=1 |
| EC: 1.1.1.38 | up | A0A2S6HU18 | Malate dehydrogenase (Oxaloacetate-decarboxylating) OS=Bacteroides xylanolyticus OX=384636 GN=BXY41_104140 PE=4 SV=1 |
|  | up | A0A0L6ZCR1 | NAD-dependent malic enzyme OS=Clostridium homopropionicum DSM 5847 OX=1121318 GN=CLHOM_08980 PE=3 SV=1 |
| EC: 1.1.1.40 | up | A0A1M6YX31 | Allosteric NADP-dependent malic enzyme OS=Prevotella ruminicola OX=839 GN=SAMN05216463_13411 PE=4 SV=1 |
| EC: 2.3.1.8 | up | A6NQH0 | Phosphate acetyltransferase OS=Pseudoflavonifractor capillosus ATCC 29799 OX=411467 GN=pta PE=4 SV=1 |
| EC: 2.7.2.1 | up | A0A412AV71 | Acetate kinase OS=[Clostridium] leptum OX=1535 GN=ackA PE=3 SV=1 |
| EC: 2.3.1.9 | up | A0A352RP61 | Acetyl-CoA C-acetyltransferase OS=Oscillibacter sp. OX=1945593 GN=DC027_06405 PE=3 SV=1 |
| EC: 4.2.1.2 | down | A0A318I1Y4 | Fumarate hydratase class I OS=Prevotella shahii DSM 15611 = JCM 12083 OX=1122991 GN=EJ73_00064 PE=3 SV=1 |
