# Supplementary material for: Dietary Supplementation With Creatine Pyruvate Alters Rumen Microbiota Protein Function in Heat-Stressed Beef Cattle
Source: Front Microbiol. 2021 Aug 27;12:715088. doi: 10.3389/fmicb.2021.715088 (PMC8431830; doi:10.3389/fmicb.2021.715088)
Supplement: Supplementary file 10 [file Table_7.DOC]

**Table S7.** Protein identity and regulation involved in glycolysis / gluconeogenesis pathway in rumen fluid samples of beef cattle fed with a CrPyr supplementation diet

| EC number | Regulate | Accession | Description |
| --- | --- | --- | --- |
| EC: 1.2.7.1 | up | A0A415ZEP4 | Pyruvate:ferredoxin (Flavodoxin) oxidoreductase OS=Butyricicoccus sp. AM05-1 OX=2292004 GN=nifJ PE=3 SV=1 |
|  |  | R6P8M9 | Pyruvate-flavodoxin oxidoreductase OS=Eubacterium sp. CAG:274 OX=1262888 GN=BN582_00957 PE=3 SV=1 |
|  |  | A0A1F8V698 | Pyruvate:ferredoxin (Flavodoxin) oxidoreductase OS=Clostridiales bacterium GWF2_38_85 OX=1797683 GN=A2Y17_09250 PE=3 SV=1 |
|  |  | A0A1Y4WGD6 | Pyruvate:ferredoxin (Flavodoxin) oxidoreductase OS=Flavonifractor sp. An100 OX=1965538 GN=B5E43_03235 PE=3 SV=1 |
|  |  | A0A166TFP4 | Pyruvate-flavodoxin oxidoreductase OS=Clostridium coskatii OX=1705578 GN=nifJ_2 PE=3 SV=1 |
|  |  | A0A1K2BLI4 | Pyruvate-ferredoxin/flavodoxin oxidoreductase OS=Ruminococcus flavefaciens OX=1265 GN=SAMN04487832_1192 PE=3 SV=1 |
|  |  | A0A353YZZ4 | Pyruvate:ferredoxin (Flavodoxin) oxidoreductase (Fragment) OS=Bacteroidales bacterium OX=2030927 GN=nifJ PE=4 SV=1 |
|  |  | A0A4D7APF0 | Pyruvate:ferredoxin (Flavodoxin) oxidoreductase OS=Dysosmobacter welbionis OX=2093857 GN=nifJ PE=3 SV=1 |
|  |  | A0A3D4RN83 | Pyruvate:ferredoxin (Flavodoxin) oxidoreductase (Fragment) OS=Bacteroidales bacterium OX=2030927 GN=nifJ PE=4 SV=1 |
|  |  | T0N108 | Pyruvate-flavodoxin oxidoreductase OS=Clostridium sp. BL8 OX=1354301 GN=M918_03780 PE=3 SV=1 |
|  |  | A0A1C5XP50 | Pyruvate synthase subunit porA OS=uncultured Flavonifractor sp. OX=1193534 GN=porA_1 PE=3 SV=1 |
|  |  | A0A1Q6SJ12 | Pyruvate:ferredoxin (Flavodoxin) oxidoreductase OS=Roseburia intestinalis OX=166486 GN=BHW46_03145 PE=3 SV=1 |
|  |  | A0A2N6AJV8 | Pyruvate:ferredoxin (Flavodoxin) oxidoreductase OS=Clostridiales bacterium OX=1898207 GN=nifJ PE=3 SV=1 |
|  | down | A0A143XWN4 | Pyruvate-flavodoxin oxidoreductase OS=Eubacteriaceae bacterium CHKCI004 OX=1780380 GN=nifJ PE=3 SV=1 |
|  |  | A0A3A9BCX9 | Pyruvate:ferredoxin (Flavodoxin) oxidoreductase (Fragment) OS=Bacteroides caecimuris OX=1796613 GN=D7W50_10630 PE=4 SV=1 |
|  |  | A0A5B7THW7 | Pyruvate:ferredoxin (Flavodoxin) oxidoreductase OS=Caloramator sp. E03 OX=2576307 GN=nifJ PE=3 SV=1 |
|  |  | A0A0K8J7N6 | Pyruvate-flavodoxin oxidoreductase OS=Herbinix luporum OX=1679721 GN=nifJ2 PE=3 SV=1 |
| EC: 1.2.7.11 | up | E6SNS3 | Pyruvate flavodoxin/ferredoxin oxidoreductase domain protein OS=Bacteroides helcogenes (strain ATCC 35417 / DSM 20613 / JCM 6297 / P 36-108) OX=693979 GN=Bache_2871 PE=4 SV=1 |
|  |  | A0A1I0PQC5 | 2-oxoglutarate ferredoxin oxidoreductase subunit alpha OS=Prevotella sp. khp7 OX=1761885 GN=SAMN04487827_1930 PE=4 SV=1 |
|  |  | A0A1I5HE68 | 2-oxoglutarate ferredoxin oxidoreductase subunit beta OS=Prevotella sp. tf2-5 OX=1761889 GN=SAMN04487852_101322 PE=4 SV=1 |
| EC: 4.1.1.49 | up | A0A1I3ZPE6 | Phosphoenolpyruvate carboxykinase (ATP) OS=Lachnospiraceae bacterium KH1T2 OX=1855374 GN=pckA PE=3 SV=1 |
|  |  | A0A255SS06 | Phosphoenolpyruvate carboxykinase (ATP) OS=Prevotella bryantii OX=77095 GN=pckA PE=3 SV=1 |
|  |  | A0A415MJA9 | Phosphoenolpyruvate carboxykinase (ATP) OS=Parabacteroides distasonis OX=823 GN=pckA PE=3 SV=1 |
|  |  | A0A239QZC1 | Phosphoenolpyruvate carboxykinase (ATP) OS=Prevotellaceae bacterium MN60 OX=1945887 GN=pckA PE=3 SV=1 |
|  |  | A0A1I5HGW5 | Phosphoenolpyruvate carboxykinase (ATP) OS=Prevotella sp. tf2-5 OX=1761889 GN=pckA PE=3 SV=1 |
|  |  | A0A355XQD4 | Phosphoenolpyruvate carboxykinase (ATP) (Fragment) OS=Parabacteroides distasonis OX=823 GN=pckA PE=3 SV=1 |
|  |  | A0A1H7IVN8 | Phosphoenolpyruvate carboxykinase (ATP) OS=Pseudobutyrivibrio ruminis OX=46206 GN=pckA PE=3 SV=1 |
|  | down | A0A355VYP5 | Phosphoenolpyruvate carboxykinase (ATP) OS=Lachnospiraceae bacterium OX=1898203 GN=pckA PE=3 SV=1 |
|  |  | A0A432LL30 | Phosphoenolpyruvate carboxykinase (ATP) OS=Prevotella sp. KCOM 3155 OX=2490854 GN=pckA PE=3 SV=1 |
|  |  | A0A316N6J8 | Phosphoenolpyruvate carboxykinase (ATP) OS=Clostridiaceae bacterium OX=1898204 GN=pckA PE=3 SV=1 |
| EC: 4.2.1.11 | up | A0A143WY68 | Enolase OS=Clostridiales bacterium CHKCI006 OX=1780379 GN=eno PE=3 SV=1 |
|  |  | A0A1C7GM69 | Enolase OS=Hungateiclostridiaceae bacterium KB18 OX=1834198 GN=eno PE=3 SV=1 |
|  |  | A0A3D0HWW4 | Enolase OS=Bacteroidales bacterium OX=2030927 GN=eno PE=3 SV=1 |
|  |  | A0A1I0MFP3 | Enolase OS=Prevotella sp. khp7 OX=1761885 GN=eno PE=3 SV=1 |
|  | down | A0A351XZ72 | Enolase OS=Porphyromonadaceae bacterium OX=2049046 GN=eno PE=3 SV=1 |
|  |  | A0A3B9TB71 | Enolase OS=Bacteroidales bacterium OX=2030927 GN=eno PE=3 SV=1 |
|  |  | A0A386XGF3 | Enolase OS=Ethanoligenens harbinense OX=253239 GN=eno PE=3 SV=1 |
|  |  | A0A1C5ZLW0 | Enolase OS=uncultured Clostridium sp. OX=59620 GN=eno PE=3 SV=1 |
| EC: 5.4.2.12 | up | A0A1H4B871 | 2,3-bisphosphoglycerate-independent phosphoglycerate mutase OS=Prevotella sp. tc2-28 OX=1761888 GN=gpmI PE=3 SV=1 |
| EC: 2.7.2.3  EC: 5.3.1.1 | up | A0A1B1YMJ8 | Multifunctional fusion protein OS=Thermoclostridium stercorarium subsp. leptospartum DSM 9219 OX=1346611 GN=tpiA PE=3 SV=1 |
| EC: 2.7.2.3 | up | A0A1C5UH45 | Phosphoglycerate kinase OS=uncultured Clostridium sp. OX=59620 GN=pgk PE=3 SV=1 |
|  |  | A0A496KMU9 | Phosphoglycerate kinase OS=Alloprevotella sp. OX=1872471 GN=pgk PE=3 SV=1 |
|  |  | A0A1I5HV96 | Phosphoglycerate kinase OS=Prevotella sp. tf2-5 OX=1761889 GN=pgk PE=3 SV=1 |
|  |  | A0A0A2TJI3 | Phosphoglycerate kinase OS=Desulfosporosinus sp. Tol-M OX=1536651 GN=pgk PE=3 SV=1 |
|  |  | A0A349QS61 | Phosphoglycerate kinase OS=Roseburia sp. OX=2049040 GN=pgk PE=3 SV=1 |
|  | down | R6XUK4 | Phosphoglycerate kinase OS=Prevotella sp. CAG:732 OX=1262934 GN=pgk PE=3 SV=1 |
|  |  | R7H8N5 | Phosphoglycerate kinase OS=Eubacterium sp. CAG:38 OX=1262889 GN=pgk PE=3 SV=1 |
|  |  | A0A357APE9 | Phosphoglycerate kinase OS=Lachnospiraceae bacterium OX=1898203 GN=pgk PE=3 SV=1 |
| EC: 1.2.1.12 | up | A0A096AYW1 | Glyceraldehyde-3-phosphate dehydrogenase OS=Prevotella amnii DNF00058 OX=1401066 GN=HMPREF9302_05785 PE=3 SV=1 |
|  |  | A0A1H3E4X2 | Glyceraldehyde-3-phosphate dehydrogenase OS=Ruminococcaceae bacterium YAD3003 OX=1520816 GN=SAMN02910264_00536 PE=3 SV=1 |
|  |  | A0A134BQI8 | Glyceraldehyde-3-phosphate dehydrogenase OS=Prevotella sp. DNF00663 OX=1384078 GN=HMPREF3034_01759 PE=3 SV=1 |
|  |  | A0A2J8B889 | Glyceraldehyde-3-phosphate dehydrogenase OS=Lachnospiraceae bacterium OX=1898203 GN=B6K86_02760 PE=3 SV=1 |
|  |  | A0A1W2DAQ5 | Glyceraldehyde-3-phosphate dehydrogenase OS=Clostridiales bacterium OX=1898207 GN=SAMN06297397_0155 PE=3 SV=1 |
|  |  | A0A1H7NIE5 | Glyceraldehyde-3-phosphate dehydrogenase OS=Ruminococcus albus OX=1264 GN=SAMN05216469_11541 PE=3 SV=1 |
|  |  | A0A1G6CDG0 | Glyceraldehyde-3-phosphate dehydrogenase OS=Ruminococcaceae bacterium FB2012 OX=1520817 GN=SAMN02910317_01537 PE=3 SV=1 |
|  |  | R9JTR3 | Glyceraldehyde-3-phosphate dehydrogenase OS=Lachnospiraceae bacterium M18-1 OX=1235792 GN=C808_03273 PE=3 SV=1 |
|  |  | A0A3B9IA35 | Type I glyceraldehyde-3-phosphate dehydrogenase (Fragment) OS=Lachnospiraceae bacterium OX=1898203 GN=DCG37_03820 PE=3 SV=1 |
|  |  | A0A1G9G7Q6 | Glyceraldehyde-3-phosphate dehydrogenase OS=Sarcina sp. DSM 11001 OX=1798184 GN=SAMN04487833_11021 PE=3 SV=1 |
|  |  | U2CHA3 | Glyceraldehyde-3-phosphate dehydrogenase OS=Clostridiales bacterium oral taxon 876 str. F0540 OX=1321778 GN=HMPREF1982_04160 PE=3 SV=1 |
|  |  | A0A3A6H7Z9 | Glyceraldehyde-3-phosphate dehydrogenase OS=Lachnospiraceae bacterium TF09-5 OX=2302969 GN=gap PE=3 SV=1 |
|  |  | A0A417U4A7 | Glyceraldehyde-3-phosphate dehydrogenase OS=Clostridium sp. OM04-12AA OX=2293041 GN=gap PE=3 SV=1 |
|  |  | A0A417GMG2 | Glyceraldehyde-3-phosphate dehydrogenase OS=Clostridium sp. AM29-11AC OX=2293028 GN=gap PE=3 SV=1 |
|  |  | R6UAX7 | Glyceraldehyde-3-phosphate dehydrogenase OS=Clostridium sp. CAG:964 OX=1262848 GN=BN818_01802 PE=3 SV=1 |
|  |  | A0A4R1N7A8 | Glyceraldehyde-3-phosphate dehydrogenase OS=Natranaerovirga hydrolytica OX=680378 GN=EDC19_0973 PE=3 SV=1 |
|  |  | A0A3C0BD03 | Glyceraldehyde-3-phosphate dehydrogenase OS=Bacteroidales bacterium OX=2030927 GN=gap PE=3 SV=1 |
|  |  | A0A3B8TYZ4 | Type I glyceraldehyde-3-phosphate dehydrogenase (Fragment) OS=Lachnospiraceae bacterium OX=1898203 GN=DCF49_01615 PE=3 SV=1 |
|  |  | A0A3D2CMN5 | Glyceraldehyde-3-phosphate dehydrogenase OS=Clostridiales bacterium OX=1898207 GN=gap PE=3 SV=1 |
|  |  | A0A417KIY7 | Glyceraldehyde-3-phosphate dehydrogenase OS=Ruminococcus sp. AM26-12LB OX=2293190 GN=gap PE=3 SV=1 |
|  |  | A0A3B9IWG2 | Type I glyceraldehyde-3-phosphate dehydrogenase (Fragment) OS=Ruminococcus sp. OX=41978 GN=DCG30_03035 PE=3 SV=1 |
|  | down | A0A1Y3WIT7 | Glyceraldehyde-3-phosphate dehydrogenase OS=Barnesiella sp. An55 OX=1965646 GN=B5G10_11265 PE=3 SV=1 |
|  |  | G5GF76 | Glyceraldehyde-3-phosphate dehydrogenase OS=Johnsonella ignava ATCC 51276 OX=679200 GN=HMPREF9333_00214 PE=3 SV=1 |
|  |  | A0A3D2N6K6 | Glyceraldehyde-3-phosphate dehydrogenase OS=Prevotella sp. OX=59823 GN=gap PE=3 SV=1 |
|  |  | I4ZAB6 | Glyceraldehyde-3-phosphate dehydrogenase OS=Prevotella bivia DSM 20514 OX=868129 GN=PrebiDRAFT_1453 PE=3 SV=1 |
|  |  | A0A2V2GJ43 | Glyceraldehyde-3-phosphate dehydrogenase OS=Ruminococcaceae bacterium OX=1898205 GN=gap PE=3 SV=1 |
|  |  | A0A2V1JPA6 | Glyceraldehyde-3-phosphate dehydrogenase OS=Eubacterium ramulus OX=39490 GN=LG34_16470 PE=3 SV=1 |
| EC: 5.3.1.1 | up | A0A1G9G839 | Triosephosphate isomerase OS=Sarcina sp. DSM 11001 OX=1798184 GN=tpiA PE=3 SV=1 |
| EC: 4.1.2.13 | up | A0A1K1N3M6 | Fructose-bisphosphate aldolase, class II OS=Ruminococcus sp. YE71 OX=244362 GN=SAMN02910447_01537 PE=4 SV=1 |
|  |  | A0A2V2GH59 | Fructose-1,6-bisphosphate aldolase, class II OS=Ruminococcaceae bacterium OX=1898205 GN=fba PE=4 SV=1 |
|  |  | A0A415GPS8 | Class II fructose-1,6-bisphosphate aldolase OS=Prevotella stercorea OX=363265 GN=DW060_03400 PE=4 SV=1 |
|  |  | A0A4Z0V464 | Class II fructose-1,6-bisphosphate aldolase OS=Duncaniella sp. TLL-A3 OX=2530391 GN=EZ315_12860 PE=4 SV=1 |
|  |  | G9YMQ1 | Fructose-1,6-bisphosphate aldolase, class II OS=Flavonifractor plautii ATCC 29863 OX=411475 GN=HMPREF0372_00773 PE=4 SV=1 |
|  |  | R5SPG0 | Fructose-1 6-bisphosphate aldolase class II OS=Bacteroides sp. CAG:661 OX=1262746 GN=BN750_00032 PE=4 SV=1 |
|  |  | R5JVR2 | Fructose-1 6-bisphosphate aldolase class II various bacterial and amitochondriate protist OS=Clostridium sp. CAG:632 OX=1262830 GN=BN743_00197 PE=4 SV=1 |
|  | down | A0A1H5WZJ4 | Fructose-bisphosphate aldolase OS=Prevotella ruminicola OX=839 GN=SAMN05216354_2591 PE=4 SV=1 |
|  |  | A0A1H3W8X0 | Fructose-bisphosphate -1aldolase, class II OS=Lachnospiraceae bacterium NK3A20 OX=877406 GN=SAMN02745687_00688 PE=4 SV=1 |
|  |  | A0A4S1ZEK8 | Class II fructose-1,6-bisphosphate aldolase OS=Bacteroidales bacterium OX=2030927 GN=E5358_09750 PE=4 SV=1 |
| EC: 2.7.1.11 | up | A0A1I5I303 | ATP-dependent 6-phosphofructokinase OS=Prevotella sp. tf2-5 OX=1761889 GN=pfkA PE=3 SV=1 |
|  |  | A0A1H8CSV5 | ATP-dependent 6-phosphofructokinase OS=Prevotella sp. ne3005 OX=1761887 GN=pfkA PE=3 SV=1 |
| EC: 2.7.1.90 | up | A0A255TC19 | Pyrophosphate--fructose 6-phosphate 1-phosphotransferase OS=Prevotella sp. P3-122 OX=2024223 GN=pfp PE=3 SV=1 |
|  |  | A0A1H0ED89 | Pyrophosphate--fructose 6-phosphate 1-phosphotransferase OS=Prevotella sp. BP1-145 OX=645273 GN=pfp PE=3 SV=1 |
|  |  | A0A4S2B5V3 | Pyrophosphate--fructose 6-phosphate 1-phosphotransferase OS=Bacteroides sp. NM69_E16B OX=2516960 GN=pfp PE=3 SV=1 |
|  |  | A0A0B2JIX5 | Pyrophosphate--fructose 6-phosphate 1-phosphotransferase OS=Coprobacter secundus OX=1501392 GN=pfp PE=3 SV=1 |
| EC: 5.3.1.9 | up | A0A0D0RBW5 | Glucose-6-phosphate isomerase OS=Lachnospiraceae bacterium TWA4 OX=1392836 GN=pgi PE=3 SV=1 |
|  |  | A0A432LJL7 | Glucose-6-phosphate isomerase OS=Prevotella sp. KCOM 3155 OX=2490854 GN=pgi PE=3 SV=1 |
|  | down | A0A1I5K9P6 | Glucose-6-phosphate isomerase OS=Prevotella sp. tf2-5 OX=1761889 GN=pgi PE=3 SV=1 |
| EC: 5.4.2.2 | up | A0A255SV21 | Phosphoglucomutase OS=Prevotella sp. P5-92 OX=2024222 GN=CIK99_08870 PE=3 SV=1 |
|  | down | A0A4S1ZK70 | Phospho-sugar mutase OS=Bacteroidales bacterium OX=2030927 GN=E5358_02215 PE=3 SV=1 |
| EC: 5.1.3.3 | up | A0A0D0I648 | Aldose 1-epimerase OS=Prevotella sp. P5-119 OX=1602171 GN=ST44_05990 PE=3 SV=1 |
|  |  | A0A1I0M8V5 | Aldose 1-epimerase OS=Prevotella sp. khp7 OX=1761885 GN=SAMN04487827_0456 PE=3 SV=1 |
|  | down | A0A1M6TZB2 | Aldose 1-epimerase OS=Prevotella ruminicola OX=839 GN=SAMN05216463_107102 PE=3 SV=1 |
| EC: 2.7.1.2 | up | A0A0M6WQR9 | Transcriptional regulator/sugar kinase OS=[Eubacterium] rectale OX=39491 GN=T1815_22051 PE=4 SV=1 |
