# Supplementary material for: Dietary Supplementation With Creatine Pyruvate Alters Rumen Microbiota Protein Function in Heat-Stressed Beef Cattle
Source: Front Microbiol. 2021 Aug 27;12:715088. doi: 10.3389/fmicb.2021.715088 (PMC8431830; doi:10.3389/fmicb.2021.715088)
Supplement: Supplementary file 11 [file Table_8.DOC]

**Table S8.** Protein identity and regulation involved in TCA cycle pathway in rumen fluid samples of beef cattle fed with a CrPyr supplementation diet

| EC number | Regulate | Accession | Description |
| --- | --- | --- | --- |
| EC: 1.2.7.1 | up | A0A415ZEP4 | Pyruvate:ferredoxin (Flavodoxin) oxidoreductase OS=Butyricicoccus sp. AM05-1 OX=2292004 GN=nifJ PE=3 SV=1 |
|  |  | R6P8M9 | Pyruvate-flavodoxin oxidoreductase OS=Eubacterium sp. CAG:274 OX=1262888 GN=BN582_00957 PE=3 SV=1 |
|  |  | A0A1F8V698 | Pyruvate:ferredoxin (Flavodoxin) oxidoreductase OS=Clostridiales bacterium GWF2_38_85 OX=1797683 GN=A2Y17_09250 PE=3 SV=1 |
|  |  | A0A1Y4WGD6 | Pyruvate:ferredoxin (Flavodoxin) oxidoreductase OS=Flavonifractor sp. An100 OX=1965538 GN=B5E43_03235 PE=3 SV=1 |
|  |  | A0A166TFP4 | Pyruvate-flavodoxin oxidoreductase OS=Clostridium coskatii OX=1705578 GN=nifJ_2 PE=3 SV=1 |
|  |  | A0A1K2BLI4 | Pyruvate-ferredoxin/flavodoxin oxidoreductase OS=Ruminococcus flavefaciens OX=1265 GN=SAMN04487832_1192 PE=3 SV=1 |
|  |  | A0A353YZZ4 | Pyruvate:ferredoxin (Flavodoxin) oxidoreductase (Fragment) OS=Bacteroidales bacterium OX=2030927 GN=nifJ PE=4 SV=1 |
|  |  | A0A4D7APF0 | Pyruvate:ferredoxin (Flavodoxin) oxidoreductase OS=Dysosmobacter welbionis OX=2093857 GN=nifJ PE=3 SV=1 |
|  |  | A0A3D4RN83 | Pyruvate:ferredoxin (Flavodoxin) oxidoreductase (Fragment) OS=Bacteroidales bacterium OX=2030927 GN=nifJ PE=4 SV=1 |
|  |  | T0N108 | Pyruvate-flavodoxin oxidoreductase OS=Clostridium sp. BL8 OX=1354301 GN=M918_03780 PE=3 SV=1 |
|  |  | A0A1C5XP50 | Pyruvate synthase subunit porA OS=uncultured Flavonifractor sp. OX=1193534 GN=porA_1 PE=3 SV=1 |
|  |  | A0A1Q6SJ12 | Pyruvate:ferredoxin (Flavodoxin) oxidoreductase OS=Roseburia intestinalis OX=166486 GN=BHW46_03145 PE=3 SV=1 |
|  |  | A0A2N6AJV8 | Pyruvate:ferredoxin (Flavodoxin) oxidoreductase OS=Clostridiales bacterium OX=1898207 GN=nifJ PE=3 SV=1 |
|  | down | A0A143XWN4 | Pyruvate-flavodoxin oxidoreductase OS=Eubacteriaceae bacterium CHKCI004 OX=1780380 GN=nifJ PE=3 SV=1 |
|  |  | A0A3A9BCX9 | Pyruvate:ferredoxin (Flavodoxin) oxidoreductase (Fragment) OS=Bacteroides caecimuris OX=1796613 GN=D7W50_10630 PE=4 SV=1 |
|  |  | A0A5B7THW7 | Pyruvate:ferredoxin (Flavodoxin) oxidoreductase OS=Caloramator sp. E03 OX=2576307 GN=nifJ PE=3 SV=1 |
|  |  | A0A0K8J7N6 | Pyruvate-flavodoxin oxidoreductase OS=Herbinix luporum OX=1679721 GN=nifJ2 PE=3 SV=1 |
| EC: 1.2.7.11 | up | E6SNS3 | Pyruvate flavodoxin/ferredoxin oxidoreductase domain protein OS=Bacteroides helcogenes (strain ATCC 35417 / DSM 20613 / JCM 6297 / P 36-108) OX=693979 GN=Bache_2871 PE=4 SV=1 |
|  |  | A0A1I0PQC5 | 2-oxoglutarate ferredoxin oxidoreductase subunit alpha OS=Prevotella sp. khp7 OX=1761885 GN=SAMN04487827_1930 PE=4 SV=1 |
|  |  | A0A1I5HE68 | 2-oxoglutarate ferredoxin oxidoreductase subunit beta OS=Prevotella sp. tf2-5 OX=1761889 GN=SAMN04487852_101322 PE=4 SV=1 |
| EC: 4.1.1.49 | up | A0A1I3ZPE6 | Phosphoenolpyruvate carboxykinase (ATP) OS=Lachnospiraceae bacterium KH1T2 OX=1855374 GN=pckA PE=3 SV=1 |
|  |  | A0A255SS06 | Phosphoenolpyruvate carboxykinase (ATP) OS=Prevotella bryantii OX=77095 GN=pckA PE=3 SV=1 |
|  |  | A0A415MJA9 | Phosphoenolpyruvate carboxykinase (ATP) OS=Parabacteroides distasonis OX=823 GN=pckA PE=3 SV=1 |
|  |  | A0A239QZC1 | Phosphoenolpyruvate carboxykinase (ATP) OS=Prevotellaceae bacterium MN60 OX=1945887 GN=pckA PE=3 SV=1 |
|  |  | A0A1I5HGW5 | Phosphoenolpyruvate carboxykinase (ATP) OS=Prevotella sp. tf2-5 OX=1761889 GN=pckA PE=3 SV=1 |
|  |  | A0A355XQD4 | Phosphoenolpyruvate carboxykinase (ATP) (Fragment) OS=Parabacteroides distasonis OX=823 GN=pckA PE=3 SV=1 |
|  |  | A0A1H7IVN8 | Phosphoenolpyruvate carboxykinase (ATP) OS=Pseudobutyrivibrio ruminis OX=46206 GN=pckA PE=3 SV=1 |
|  | down | A0A355VYP5 | Phosphoenolpyruvate carboxykinase (ATP) OS=Lachnospiraceae bacterium OX=1898203 GN=pckA PE=3 SV=1 |
|  |  | A0A432LL30 | Phosphoenolpyruvate carboxykinase (ATP) OS=Prevotella sp. KCOM 3155 OX=2490854 GN=pckA PE=3 SV=1 |
|  |  | A0A316N6J8 | Phosphoenolpyruvate carboxykinase (ATP) OS=Clostridiaceae bacterium OX=1898204 GN=pckA PE=3 SV=1 |
| EC: 6.4.1.1 | up | A0A1M6T8D3 | Pyruvate carboxylase subunit B OS=Prevotella ruminicola OX=839 GN=SAMN05216463_10566 PE=4 SV=1 |
| EC: 1.3.5.4  EC: 1.3.5.1 | up | R5I1J4 | Uncharacterized protein OS=Alistipes sp. CAG:831 OX=1262698 GN=BN796_00018 PE=4 SV=1 |
|  |  | A0A134BUM5 | Succinate dehydrogenase or fumarate reductase, flavoprotein subunit OS=Prevotella sp. DNF00663 OX=1384078 GN=HMPREF3034_01027 PE=4 SV=1 |
|  |  | A0A359AUQ2 | Fumarate reductase/succinate dehydrogenase flavoprotein subunit OS=Porphyromonadaceae bacterium OX=2049046 GN=DEF88_03940 PE=4 SV=1 |
|  |  | A0A2L2WM32 | Succinate dehydrogenase/fumarate reductase iron-sulfur subunit (Fragment) OS=Prevotella sp. MGM1 OX=2033405 GN=PvtlMGM1_1394 PE=4 SV=1 |
|  |  | A0A412UAJ8 | Succinate dehydrogenase/fumarate reductase iron-sulfur subunit OS=Paraprevotella clara OX=454154 GN=DWW55_07530 PE=4 SV=1 |
|  | down | A0A2E2Y0F5 | Succinate dehydrogenase flavoprotein subunit OS=Lentimicrobiaceae bacterium OX=2026756 GN=sdhA PE=4 SV=1 |
|  |  | A0A239R145 | Succinate dehydrogenase subunit A OS=Prevotellaceae bacterium MN60 OX=1945887 GN=SAMN06298211_10697 PE=4 SV=1 |
| EC: 4.2.1.2 | down | A0A318I1Y4 | Fumarate hydratase class I OS=Prevotella shahii DSM 15611 = JCM 12083 OX=1122991 GN=EJ73_00064 PE=3 SV=1 |
