# Supplementary material for: Dietary Supplementation With Creatine Pyruvate Alters Rumen Microbiota Protein Function in Heat-Stressed Beef Cattle
Source: Front Microbiol. 2021 Aug 27;12:715088. doi: 10.3389/fmicb.2021.715088 (PMC8431830; doi:10.3389/fmicb.2021.715088)
Supplement: Supplementary file 12 [file Table_9.DOC]

**Table S9.** Protein identity and regulation involved in nitrogen metabolism pathway in rumen fluid samples of beef cattle fed with a CrPyr supplementation diet

| EC number | Regulate | Accession | Description |
| --- | --- | --- | --- |
| EC: 1.4.1.4 | up | A0A415DYQ2 | Glutamate dehydrogenase OS=Emergencia timonensis OX=1776384 GN=DW099_14140 PE=3 SV=1 |
|  |  | G4L1F1 | Glutamate dehydrogenase OS=Oscillibacter valericigenes (strain DSM 18026 / NBRC 101213 / Sjm18-20) OX=693746 GN=gdh PE=3 SV=1 |
|  |  | A0A4Q5JM31 | Glutamate dehydrogenase OS=Alistipes finegoldii OX=214856 GN=EAI98_09700 PE=3 SV=1 |
|  |  | E0NVC5 | Glutamate dehydrogenase OS=Prevotella marshii DSM 16973 = JCM 13450 OX=862515 GN=gdh PE=3 SV=1 |
|  |  | R5PD20 | Glutamate dehydrogenase OS=Odoribacter sp. CAG:788 OX=1262909 GN=BN783_02989 PE=3 SV=1 |
|  |  | A0A3P1Z3F0 | NADP-specific glutamate dehydrogenase OS=Tannerella forsythia OX=28112 GN=EII40_06340 PE=3 SV=1 |
|  |  | A0A3N0I160 | NADP-specific glutamate dehydrogenase OS=Clostridium sp. YH-panp20 OX=2486714 GN=EDX97_05265 PE=3 SV=1 |
|  |  | A0A2N6Q8D3 | Glutamate dehydrogenase OS=Prevotella timonensis OX=386414 GN=CJ232_00320 PE=3 SV=1 |
|  | down | A0A2V2GNA8 | Glutamate dehydrogenase OS=Ruminococcaceae bacterium OX=1898205 GN=DBX45_03510 PE=3 SV=1 |
|  |  | A0A1Q6FVH2 | Glutamate dehydrogenase OS=Bacteroides sp. CAG:1060_57_27 OX=1896975 GN=BHV78_04505 PE=3 SV=1 |
|  |  | A0A3E2SV33 | Glutamate dehydrogenase OS=Harryflintia acetispora OX=1849041 GN=DW086_09505 PE=3 SV=1 |
|  |  | A0A1H5WTH8 | Glutamate dehydrogenase OS=Butyrivibrio sp. Su6 OX=1520810 GN=SAMN02910276_01671 PE=3 SV=1 |
|  |  | A0A1J5HG21 | Glutamate dehydrogenase OS=Porphyromonadaceae bacterium CG2_30_38_12 OX=1805325 GN=AUK44_05365 PE=3 SV=1 |
|  |  | A0A1M3DNP2 | Glutamate dehydrogenase OS=Bacteroidales bacterium 36-12 OX=1895718 GN=BGO29_05435 PE=3 SV=1 |
|  |  | A0A1G7PVR8 | Glutamate dehydrogenase OS=Bacteroidales bacterium KHT7 OX=1855373 GN=SAMN05216518_11523 PE=3 SV=1 |
|  |  | A0A0B0BUT2 | Glutamate dehydrogenase OS=Alistipes inops OX=1501391 GN=LG35_09060 PE=3 SV=1 |
|  |  | A0A3E2B190 | Glutamate dehydrogenase OS=Evtepia gabavorous OX=2211183 GN=DV520_10995 PE=3 SV=1 |
|  |  | A0A1H8EGD0 | Glutamate dehydrogenase OS=Prevotella sp. ne3005 OX=1761887 GN=SAMN04487902_10878 PE=3 SV=1 |
|  |  | A0A353HCL7 | Glutamate dehydrogenase OS=Clostridiales bacterium OX=1898207 GN=DDW16_03920 PE=3 SV=1 |
|  |  | E6SRN3 | Glutamate dehydrogenase OS=Bacteroides helcogenes (strain ATCC 35417 / DSM 20613 / JCM 6297 / P 36-108) OX=693979 GN=Bache_3199 PE=3 SV=1 |
|  |  | E4T0X1 | Glutamate dehydrogenase OS=Paludibacter propionicigenes (strain DSM 17365 / JCM 13257 / WB4) OX=694427 GN=Palpr_0190 PE=3 SV=1 |
| EC: 6.3.1.2 | up | A0A1H4BL18 | Glutamine synthetase OS=Prevotella sp. tc2-28 OX=1761888 GN=SAMN04487851_10737 PE=3 SV=1 |
|  |  | A0A174D0Q4 | Glutamine synthetase OS=Blautia obeum OX=40520 GN=glnA_2 PE=3 SV=1 |
|  |  | A0A3D3RLB9 | Glutamine synthetase type III OS=Lachnospiraceae bacterium OX=1898203 GN=DIT54_03125 PE=3 SV=1 |
|  | down | A0A1K1NRY1 | Glutamine synthetase OS=Prevotellaceae bacterium HUN156 OX=1520830 GN=SAMN02910409_1930 PE=3 SV=1 |
|  |  | R5LYR9 | Glutamine synthetase type III OS=Prevotella sp. CAG:1185 OX=1262921 GN=BN473_00821 PE=3 SV=1 |
|  |  | A0A1H0D434 | Glutamine synthetase OS=Prevotella sp. BP1-145 OX=645273 GN=SAMN04487900_101265 PE=3 SV=1 |
| EC: 1.4.1.13 | down | V8C128 | Glutamate synthase (NADPH), homotetrameric OS=Prevotella oralis CC98A OX=1073367 GN=HMPREF1199_00161 PE=4 SV=1 |

**Table S10.** Protein identity and regulation involved in biosynthesis of amino acid pathway in rumen fluid samples of beef cattle fed with a CrPyr supplementation diet

| EC number | Regulate | Accession | Description |
| --- | --- | --- | --- |
| EC: 2.7.1.11 | up | A0A1I5I303 | ATP-dependent 6-phosphofructokinase OS=Prevotella sp. tf2-5 OX=1761889 GN=pfkA PE=3 SV=1 |
|  |  | A0A1H8CSV5 | ATP-dependent 6-phosphofructokinase OS=Prevotella sp. ne3005 OX=1761887 GN=pfkA PE=3 SV=1 |
| EC: 4.1.2.13 | up | A0A1K1N3M6 | Fructose-bisphosphate aldolase, class II OS=Ruminococcus sp. YE71 OX=244362 GN=SAMN02910447_01537 PE=4 SV=1 |
|  |  | A0A2V2GH59 | Fructose-1,6-bisphosphate aldolase, class II OS=Ruminococcaceae bacterium OX=1898205 GN=fba PE=4 SV=1 |
|  |  | A0A415GPS8 | Class II fructose-1,6-bisphosphate aldolase OS=Prevotella stercorea OX=363265 GN=DW060_03400 PE=4 SV=1 |
|  |  | A0A4Z0V464 | Class II fructose-1,6-bisphosphate aldolase OS=Duncaniella sp. TLL-A3 OX=2530391 GN=EZ315_12860 PE=4 SV=1 |
|  |  | G9YMQ1 | Fructose-1,6-bisphosphate aldolase, class II OS=Flavonifractor plautii ATCC 29863 OX=411475 GN=HMPREF0372_00773 PE=4 SV=1 |
|  |  | R5SPG0 | Fructose-1 6-bisphosphate aldolase class II OS=Bacteroides sp. CAG:661 OX=1262746 GN=BN750_00032 PE=4 SV=1 |
|  |  | R5JVR2 | Fructose-1 6-bisphosphate aldolase class II various bacterial and amitochondriate protist OS=Clostridium sp. CAG:632 OX=1262830 GN=BN743_00197 PE=4 SV=1 |
|  | down | A0A1H5WZJ4 | Fructose-bisphosphate aldolase OS=Prevotella ruminicola OX=839 GN=SAMN05216354_2591 PE=4 SV=1 |
|  |  | A0A1H3W8X0 | Fructose-bisphosphate aldolase, class II OS=Lachnospiraceae bacterium NK3A20 OX=877406 GN=SAMN02745687_00688 PE=4 SV=1 |
|  |  | A0A4S1ZEK8 | Class II fructose-1,6-bisphosphate aldolase OS=Bacteroidales bacterium OX=2030927 GN=E5358_09750 PE=4 SV=1 |
| EC: 5.3.1.1 | up | A0A1B1YMJ8 | Multifunctional fusion protein OS=Thermoclostridium stercorarium subsp. leptospartum DSM 9219 OX=1346611 GN=tpiA PE=3 SV=1 |
|  |  | A0A1G9G839 | Triosephosphate isomerase OS=Sarcina sp. DSM 11001 OX=1798184 GN=tpiA PE=3 SV=1 |
| EC: 1.2.1.12 | up | A0A096AYW1 | Glyceraldehyde-3-phosphate dehydrogenase OS=Prevotella amnii DNF00058 OX=1401066 GN=HMPREF9302_05785 PE=3 SV=1 |
|  |  | A0A1H3E4X2 | Glyceraldehyde-3-phosphate dehydrogenase OS=Ruminococcaceae bacterium YAD3003 OX=1520816 GN=SAMN02910264_00536 PE=3 SV=1 |
|  |  | A0A134BQI8 | Glyceraldehyde-3-phosphate dehydrogenase OS=Prevotella sp. DNF00663 OX=1384078 GN=HMPREF3034_01759 PE=3 SV=1 |
|  |  | A0A2J8B889 | Glyceraldehyde-3-phosphate dehydrogenase OS=Lachnospiraceae bacterium OX=1898203 GN=B6K86_02760 PE=3 SV=1 |
|  |  | A0A1W2DAQ5 | Glyceraldehyde-3-phosphate dehydrogenase OS=Clostridiales bacterium OX=1898207 GN=SAMN06297397_0155 PE=3 SV=1 |
|  |  | A0A1H7NIE5 | Glyceraldehyde-3-phosphate dehydrogenase OS=Ruminococcus albus OX=1264 GN=SAMN05216469_11541 PE=3 SV=1 |
|  |  | A0A1G6CDG0 | Glyceraldehyde-3-phosphate dehydrogenase OS=Ruminococcaceae bacterium FB2012 OX=1520817 GN=SAMN02910317_01537 PE=3 SV=1 |
|  |  | R9JTR3 | Glyceraldehyde-3-phosphate dehydrogenase OS=Lachnospiraceae bacterium M18-1 OX=1235792 GN=C808_03273 PE=3 SV=1 |
|  |  | A0A3B9IA35 | Type I glyceraldehyde-3-phosphate dehydrogenase (Fragment) OS=Lachnospiraceae bacterium OX=1898203 GN=DCG37_03820 PE=3 SV=1 |
|  |  | A0A1G9G7Q6 | Glyceraldehyde-3-phosphate dehydrogenase OS=Sarcina sp. DSM 11001 OX=1798184 GN=SAMN04487833_11021 PE=3 SV=1 |
|  |  | U2CHA3 | Glyceraldehyde-3-phosphate dehydrogenase OS=Clostridiales bacterium oral taxon 876 str. F0540 OX=1321778 GN=HMPREF1982_04160 PE=3 SV=1 |
|  |  | A0A3A6H7Z9 | Glyceraldehyde-3-phosphate dehydrogenase OS=Lachnospiraceae bacterium TF09-5 OX=2302969 GN=gap PE=3 SV=1 |
|  |  | A0A417U4A7 | Glyceraldehyde-3-phosphate dehydrogenase OS=Clostridium sp. OM04-12AA OX=2293041 GN=gap PE=3 SV=1 |
|  |  | A0A417GMG2 | Glyceraldehyde-3-phosphate dehydrogenase OS=Clostridium sp. AM29-11AC OX=2293028 GN=gap PE=3 SV=1 |
|  |  | R6UAX7 | Glyceraldehyde-3-phosphate dehydrogenase OS=Clostridium sp. CAG:964 OX=1262848 GN=BN818_01802 PE=3 SV=1 |
|  |  | A0A4R1N7A8 | Glyceraldehyde-3-phosphate dehydrogenase OS=Natranaerovirga hydrolytica OX=680378 GN=EDC19_0973 PE=3 SV=1 |
|  |  | A0A3C0BD03 | Glyceraldehyde-3-phosphate dehydrogenase OS=Bacteroidales bacterium OX=2030927 GN=gap PE=3 SV=1 |
|  |  | A0A3B8TYZ4 | Type I glyceraldehyde-3-phosphate dehydrogenase (Fragment) OS=Lachnospiraceae bacterium OX=1898203 GN=DCF49_01615 PE=3 SV=1 |
|  |  | A0A3D2CMN5 | Glyceraldehyde-3-phosphate dehydrogenase OS=Clostridiales bacterium OX=1898207 GN=gap PE=3 SV=1 |
|  |  | A0A417KIY7 | Glyceraldehyde-3-phosphate dehydrogenase OS=Ruminococcus sp. AM26-12LB OX=2293190 GN=gap PE=3 SV=1 |
|  |  | A0A3B9IWG2 | Type I glyceraldehyde-3-phosphate dehydrogenase (Fragment) OS=Ruminococcus sp. OX=41978 GN=DCG30_03035 PE=3 SV=1 |
|  | down | A0A1Y3WIT7 | Glyceraldehyde-3-phosphate dehydrogenase OS=Barnesiella sp. An55 OX=1965646 GN=B5G10_11265 PE=3 SV=1 |
|  |  | G5GF76 | Glyceraldehyde-3-phosphate dehydrogenase OS=Johnsonella ignava ATCC 51276 OX=679200 GN=HMPREF9333_00214 PE=3 SV=1 |
|  |  | A0A3D2N6K6 | Glyceraldehyde-3-phosphate dehydrogenase OS=Prevotella sp. OX=59823 GN=gap PE=3 SV=1 |
|  |  | I4ZAB6 | Glyceraldehyde-3-phosphate dehydrogenase OS=Prevotella bivia DSM 20514 OX=868129 GN=PrebiDRAFT_1453 PE=3 SV=1 |
|  |  | A0A2V2GJ43 | Glyceraldehyde-3-phosphate dehydrogenase OS=Ruminococcaceae bacterium OX=1898205 GN=gap PE=3 SV=1 |
|  |  | A0A2V1JPA6 | Glyceraldehyde-3-phosphate dehydrogenase OS=Eubacterium ramulus OX=39490 GN=LG34_16470 PE=3 SV=1 |
| EC: 2.7.2.3 | up | A0A1B1YMJ8 | Multifunctional fusion protein OS=Thermoclostridium stercorarium subsp. leptospartum DSM 9219 OX=1346611 GN=tpiA PE=3 SV=1 |
|  |  | A0A1C5UH45 | Phosphoglycerate kinase OS=uncultured Clostridium sp. OX=59620 GN=pgk PE=3 SV=1 |
|  |  | A0A496KMU9 | Phosphoglycerate kinase OS=Alloprevotella sp. OX=1872471 GN=pgk PE=3 SV=1 |
|  |  | A0A1I5HV96 | Phosphoglycerate kinase OS=Prevotella sp. tf2-5 OX=1761889 GN=pgk PE=3 SV=1 |
|  |  | A0A0A2TJI3 | Phosphoglycerate kinase OS=Desulfosporosinus sp. Tol-M OX=1536651 GN=pgk PE=3 SV=1 |
|  |  | A0A349QS61 | Phosphoglycerate kinase OS=Roseburia sp. OX=2049040 GN=pgk PE=3 SV=1 |
|  | down | R6XUK4 | Phosphoglycerate kinase OS=Prevotella sp. CAG:732 OX=1262934 GN=pgk PE=3 SV=1 |
|  |  | R7H8N5 | Phosphoglycerate kinase OS=Eubacterium sp. CAG:38 OX=1262889 GN=pgk PE=3 SV=1 |
|  |  | A0A357APE9 | Phosphoglycerate kinase OS=Lachnospiraceae bacterium OX=1898203 GN=pgk PE=3 SV=1 |
| EC: 5.4.2.12 | up | A0A1H4B871 | 2,3-bisphosphoglycerate-independent phosphoglycerate mutase OS=Prevotella sp. tc2-28 OX=1761888 GN=gpmI PE=3 SV=1 |
| EC: 4.2.1.11 | up | A0A143WY68 | Enolase OS=Clostridiales bacterium CHKCI006 OX=1780379 GN=eno PE=3 SV=1 |
|  |  | A0A1C7GM69 | Enolase OS=Hungateiclostridiaceae bacterium KB18 OX=1834198 GN=eno PE=3 SV=1 |
|  |  | A0A3D0HWW4 | Enolase OS=Bacteroidales bacterium OX=2030927 GN=eno PE=3 SV=1 |
|  |  | A0A1I0MFP3 | Enolase OS=Prevotella sp. khp7 OX=1761885 GN=eno PE=3 SV=1 |
|  | down | A0A351XZ72 | Enolase OS=Porphyromonadaceae bacterium OX=2049046 GN=eno PE=3 SV=1 |
|  |  | A0A3B9TB71 | Enolase OS=Bacteroidales bacterium OX=2030927 GN=eno PE=3 SV=1 |
|  |  | A0A386XGF3 | Enolase OS=Ethanoligenens harbinense OX=253239 GN=eno PE=3 SV=1 |
|  |  | A0A1C5ZLW0 | Enolase OS=uncultured Clostridium sp. OX=59620 GN=eno PE=3 SV=1 |
| EC: 6.4.1.1 | up | A0A1M6T8D3 | Pyruvate carboxylase subunit B OS=Prevotella ruminicola OX=839 GN=SAMN05216463_10566 PE=4 SV=1 |
| EC: 4.2.1.19  EC: 3.1.3.15 | up | A0A1I0M8P1 | Histidine biosynthesis bifunctional protein HisB OS=Prevotella sp. khp7 OX=1761885 GN=hisB PE=3 SV=1 |
| EC: 2.7.6.1 | up | H1Q2P2 | Ribose-phosphate pyrophosphokinase OS=Prevotella micans F0438 OX=883158 GN=HMPREF9140_01180 PE=4 SV=1 |
|  | down | A0A133XU45 | Ribose-phosphate diphosphokinase OS=Bacteroidales bacterium KA00251 OX=1497953 GN=HMPREF1869_01181 PE=4 SV=1 |
| EC: 2.2.1.1 | up | A0A1I5KJE9 | Transketolase OS=Prevotella sp. tf2-5 OX=1761889 GN=SAMN04487852_10955 PE=3 SV=1 |
|  |  | A0A1G7UL00 | Transketolase OS=Prevotella sp. BP1-148 OX=645274 GN=SAMN04487901_104163 PE=3 SV=1 |
| EC: 4.2.1.20 | down | A0A1H8DH72 | Tryptophan synthase OS=Prevotella sp. ne3005 OX=1761887 GN=SAMN04487902_106206 PE=3 SV=1 |
|  |  | A0A2N6QQV4 | Tryptophan synthase OS=Prevotella buccalis OX=28127 GN=CJ231_07005 PE=3 SV=1 |
| EC: 1.1.1.95 | down | A0A2L2WSG2 | 3-phosphoglycerate dehydrogenase OS=Prevotella sp. MGM1 OX=2033405 GN=PvtlMGM1_1843 PE=3 SV=1 |
| EC: 2.6.1.52 | up | A0A356PW04 | Phosphoserine aminotransferase OS=Ruminococcaceae bacterium OX=1898205 GN=serC PE=3 SV=1 |
|  |  | A0A373TM38 | Phosphoserine aminotransferase OS=Clostridium sp. AF23-6LB OX=2293005 GN=serC PE=3 SV=1 |
|  |  | A0A1H8A2D5 | Phosphoserine aminotransferase OS=Prevotella sp. ne3005 OX=1761887 GN=serC PE=3 SV=1 |
|  | down | A0A1G7Z3E0 | Phosphoserine aminotransferase OS=Prevotella sp. BP1-148 OX=645274 GN=serC PE=3 SV=1 |
|  |  | A0A3N5E1M1 | Phosphoserine aminotransferase OS=Bacteroidales bacterium OX=2030927 GN=serC PE=3 SV=1 |
| EC: 4.3.1.17 | up | A0A1F0G755 | Serine dehydratase OS=Porphyromonas sp. HMSC077F02 OX=1739529 GN=HMPREF3027_02200 PE=4 SV=1 |
| EC: 2.5.1.47 | up | A0A1H4ALT9 | Cysteine synthase OS=Prevotella sp. tc2-28 OX=1761888 GN=SAMN04487851_10571 PE=3 SV=1 |
| EC: 2.1.1.13 | up | U2DNY0 | Putative dimethylamine corrinoid protein OS=Blautia sp. KLE 1732 OX=1226324 GN=HMPREF1547_00077 PE=4 SV=1 |
| EC: 2.1.2.1 | down | A0A3D3FV79 | Serine hydroxymethyltransferase OS=Bacteroidales bacterium OX=2030927 GN=glyA PE=3 SV=1 |
|  |  | A0A1M6Q648 | Serine hydroxymethyltransferase OS=Hathewaya proteolytica DSM 3090 OX=1121331 GN=glyA PE=3 SV=1 |
|  |  | A0A350PN74 | Serine hydroxymethyltransferase OS=Prevotella sp. OX=59823 GN=glyA PE=3 SV=1 |
| EC: 1.1.1.3  EC: 2.7.2.4 | down | A0A1I0LYR8 | Aspartate kinase OS=Prevotella aff. ruminicola Tc2-24 OX=81582 GN=SAMN04487850_0102 PE=4 SV=1 |
| EC: 2.7.2.4 | down | A0A1I0M1Q4 | Aspartokinase OS=Prevotella aff. ruminicola Tc2-24 OX=81582 GN=SAMN04487850_0161 PE=3 SV=1 |
| EC: 6.3.5.4 | down | A0A380YLK4 | Asparagine synthase OS=Bacteroides eggerthii OX=28111 GN=asnB_2 PE=4 SV=1 |
| EC: 1.2.1.11 | up | A0A1C6BEK6 | Aspartate-semialdehyde dehydrogenase OS=uncultured Flavonifractor sp. OX=1193534 GN=asd PE=3 SV=1 |
| EC: 1.1.1.86 | down | A0A1I5I3W8 | Ketol-acid reductoisomerase (NADP(+)) OS=Prevotella sp. tf2-5 OX=1761889 GN=SAMN04487852_102335 PE=3 SV=1 |
|  |  | R6SJY5 | Ketol-acid reductoisomerase (NADP(+)) OS=Bacteroides coprophilus CAG:333 OX=1263041 GN=BN612_00620 PE=3 SV=1 |
| EC: 2.6.1.42 | down | A0A1H3WW34 | Branched-chain amino acid aminotransferase OS=Prevotella sp. tc2-28 OX=1761888 GN=SAMN04487851_10182 PE=4 SV=1 |
| EC: 6.3.1.2 | up | A0A1H4BL18 | Glutamine synthetase OS=Prevotella sp. tc2-28 OX=1761888 GN=SAMN04487851_10737 PE=3 SV=1 |
|  |  | A0A174D0Q4 | Glutamine synthetase OS=Blautia obeum OX=40520 GN=glnA_2 PE=3 SV=1 |
|  |  | A0A3D3RLB9 | Glutamine synthetase type III OS=Lachnospiraceae bacterium OX=1898203 GN=DIT54_03125 PE=3 SV=1 |
|  | down | A0A1K1NRY1 | Glutamine synthetase OS=Prevotellaceae bacterium HUN156 OX=1520830 GN=SAMN02910409_1930 PE=3 SV=1 |
|  |  | R5LYR9 | Glutamine synthetase type III OS=Prevotella sp. CAG:1185 OX=1262921 GN=BN473_00821 PE=3 SV=1 |
|  |  | A0A1H0D434 | Glutamine synthetase OS=Prevotella sp. BP1-145 OX=645273 GN=SAMN04487900_101265 PE=3 SV=1 |
| EC: 1.4.1.13 | down | V8C128 | Glutamate synthase (NADPH), homotetrameric OS=Prevotella oralis CC98A OX=1073367 GN=HMPREF1199_00161 PE=4 SV=1 |
| EC: 6.3.4.5 | up | A0A2V2E1D2 | Argininosuccinate synthase OS=Clostridiales bacterium OX=1898207 GN=argG PE=3 SV=1 |
|  | down | R6UBU6 | Argininosuccinate synthase OS=Clostridium sp. CAG:964 OX=1262848 GN=argG PE=3 SV=1 |
